# Multimodal driver emotion recognition using motor activity and facial expressions

**DOI:** 10.3389/frai.2024.1467051

**Published:** 2024-11-27

**Authors:** Carlos H. Espino-Salinas, Huizilopoztli Luna-García, José M. Celaya-Padilla, Cristian Barría-Huidobro, Nadia Karina Gamboa Rosales, David Rondon, Klinge Orlando Villalba-Condori

**Affiliations:** ^1^Laboratorio de Tecnologías Interactivas y Experiencia de Usuario, Universidad Autónoma de Zacatecas, Unidad Academica de Ingeniería Electrica, Zacatecas, Mexico; ^2^Centro de Investigación en Ciberseguridad, Universidad Mayor de Chile, Providencia, Chile; ^3^Departamento Estudios Generales, Universidad Continental, Arequipa, Peru; ^4^Vicerrectorado de Investigación, Catholic University of Santa María, Arequipa, Peru

**Keywords:** facial emotion recognition, motor activity, driver emotions, transfer learning, convolutional neural network, ADAS

## Abstract

Driving performance can be significantly impacted when a person experiences intense emotions behind the wheel. Research shows that emotions such as anger, sadness, agitation, and joy can increase the risk of traffic accidents. This study introduces a methodology to recognize four specific emotions using an intelligent model that processes and analyzes signals from motor activity and driver behavior, which are generated by interactions with basic driving elements, along with facial geometry images captured during emotion induction. The research applies machine learning to identify the most relevant motor activity signals for emotion recognition. Furthermore, a pre-trained Convolutional Neural Network (CNN) model is employed to extract probability vectors from images corresponding to the four emotions under investigation. These data sources are integrated through a unidimensional network for emotion classification. The main proposal of this research was to develop a multimodal intelligent model that combines motor activity signals and facial geometry images to accurately recognize four specific emotions (anger, sadness, agitation, and joy) in drivers, achieving a 96.0% accuracy in a simulated environment. The study confirmed a significant relationship between drivers' motor activity, behavior, facial geometry, and the induced emotions.

## 1 Introduction

Road accidents are among the leading causes of death worldwide, with approximately 1.3 million people losing their lives in traffic accidents each year. Additionally, 20 to 50 million individuals suffer non-fatal injuries, many of which lead to long-term disabilities. These injuries result in significant economic losses for individuals, their families, and nations as a whole (WHO, 2018). Various factors contribute to the high incidence of accidents, underscoring the need for effective interventions. Identifying these interventions requires a thorough analysis and classification of the factors that lead to accidents.

One of the most common causes of road accidents is the high cognitive load placed on drivers. They must continuously process a stream of visual information from the road, traffic signs, pedestrians, other vehicles, and the environment. Several situational factors can increase this cognitive load, including the presence of similarly aged passengers who may distract the driver, fatigue (particularly common among young drivers), and socioeconomic status, which can influence risk-taking behavior behind the wheel (Rezapour and Ksaibati, 2022).

The growing use of mobile phones has further increased the risk of accidents, especially among young drivers. The cognitive and behavioral demands of phone usage while driving divert attention from the road. Additionally, alcohol and drug consumption impairs cognitive processes and increases crash risks across all age groups (Celaya-Padilla et al., 2021). According to the National Highway Traffic Safety Administration (NHTSA), driver distraction occurs when attention shifts from driving to other activities, contributing to accidents. In 2021 alone, 3,522 people were killed, and approximately 36,241 were injured in traffic accidents caused by distracted driving. Research shows that both internal and external factors can lead to road accidents. One significant internal factor is emotional state, which can affect driving behavior and lead to erratic or inadequate driving. Emotions, which are often difficult to control, can unexpectedly influence a driver's behavior (Maldonado et al., 2020). They are transient mental states that can change rapidly in response to significant events, triggering behavioral responses that may be difficult to regulate (Zimasa et al., 2017).

Negative emotions, in particular, can significantly affect drivers. Studies have demonstrated a link between negative emotions and impaired driving performance. For example, sadness has been shown to increase location error rates, while anger slows drivers' ability to identify road elements (Dozio et al., 2024). Emotions such as anger, hostility, and nervousness are strongly associated with aggressive driving behaviors (Stephens et al., 2024). These negative emotions can impair cognitive processes and compromise road safety (Zhang Q. et al., 2020). A study by Dingus et al. (2016) found that drivers experiencing sadness, anger, or agitation were nearly ten times more likely to be involved in an accident.

Advanced Driver Assistance Systems (ADAS) must ensure safe transportation by taking into account drivers' vulnerability to accidents and recognizing that systems should be designed to accommodate human error (WHO, 2018). To assess a driver's readiness, these systems need to monitor the driver's physical, emotional, and physiological state and communicate relevant information effectively. Various real-time emotion recognition systems have been developed within the fields of affective computing and ADAS, with the goal of adapting to users' emotions for more natural and efficient interactions (Schuetz and Venkatesh, 2020). Emotion recognition allows interactive vehicle systems to interpret human emotions and use this data to make decisions. However, current ADAS largely implement basic mechanisms for emotional state recognition. If ADAS could account for a driver's emotional state, they could make more contextualized decisions based on the driver's potential reactions. Developing ADAS that continuously recognize both the driver's emotions and performance remains a significant challenge (Davoli et al., 2020). Emotion recognition has therefore become a central feature of vehicle systems, relying on various measurements–such as facial expressions, speech, gait patterns, physiology, and eye-tracking–analyzed using advanced techniques like artificial intelligence (Cai et al., 2023).

Despite the significant progress in developing less intrusive and more accurate methods for emotion recognition in automotive environments, numerous challenges remain. Traditional approaches often rely on camera-based systems, which can face issues such as occlusion, lighting variability, and differences in drivers' physical characteristics in uncontrolled environments. Additionally, control mechanisms based on biophysiological data, though potentially effective, tend to be intrusive and may cause emotional discomfort to drivers. Research into the motor activity and driving behavior of drivers, while promising, has been limited, with only a few studies utilizing basic artificial intelligence techniques to explore these characteristics.

To address the challenges of emotion recognition, a novel approach is proposed: the development of a multimodal artificial intelligence model for objective emotion recognition. This model will integrate data from motor activity and facial geometric changes to improve emotion recognition accuracy.

The remainder of this article is structured as follows: In Section 2, The most recent works in the area of emotion recognition related to the present study are mentioned. Section 3 explains the materials and methods used to generate an optimal and efficient multimodal emotion recognition model. Section 4 describes the results of the emotion induction phase and the developed emotion recognition model. In Section 5, a comprehensive discussion of the results obtained is made, emphasizing the contribution of the research to the existing body of knowledge. Finally, in Section 6, final conclusions and proposals for future research aimed at improving emotion recognition systems in drivers covering the analysis and processing of various information sources are presented.

## 2 Related studies

Related works in emotion recognition encompass a broad spectrum of research endeavors aimed at understanding and interpreting human emotions through various modalities. These works often explore the utilization of machine learning techniques, including deep learning algorithms, to detect, classify, and interpret emotional states. Some key areas of research and notable contributions.

### 2.1 Facial emotion recognition

Thanks to recent and continuous improvements in the application of artificial neural networks, many architectures have been proposed and employed for facial emotion recognition, each of which has surpassed its predecessors, thus improving the accuracy and performance of the latest generation (Ko, 2018).

Some studies propose the implementation of deep learning algorithms, such as Convolutional Neural Networks (CNNs), for facial emotion recognition. These studies often train CNNs with facial emotion data and test different architectures, including VGG-16, VGG-19, ResNet-18, ResNet-34, ResNet-50, ResNet-152, Inception-v3, and DenseNet-161, using facial image datasets. For example, Mehendale (2020), proposes a CNN-based approach for facial emotion recognition. This network consists of two stages: the first stage removes the background of the image, while the second stage focuses on extracting facial features. The two-level CNN operates in series, with the final layer of the perceptron adjusting weights and exponent values after each iteration. This approach contrasts with the single-level CNN strategies typically used and results in improved accuracy. Similarly, Sarvakar et al. (2023), classified facial expressions into one of seven emotions using various models on an emotion dataset. The models tested include decision trees, feed-forward neural networks, and CNNs, achieving reasonably acceptable accuracy. Additionally, Modi and Bohara (2021) presents a CNN-based facial expression recognition framework in which the network classifies facial expressions as happy, sad, or neutral. In a related study, Khattak et al. (2022), addresses emotion recognition by applying a deep learning technique using CNNs to classify facial emotions and detect age and gender from facial expressions. The experimental results demonstrate that the proposed model can identify emotions, age, and gender with a high degree of accuracy.

Also, some researches has made significant advancements in facial emotion recognition using multimodal models. An example is the work of (Mocanu et al., 2023), which proposes an innovative methodology that integrates simultaneous video and audio analysis. For visual analysis, they employ a three-dimensional CNN, and for auditory analysis, they use a two-dimensional CNN. This study implements the ResNet-101 and ResNet-18 architectures, achieving impressive results with an average accuracy of 89.25% on the RAVDESS database and 84.57% on the CREMA-D database. These results represent a substantial improvement over previous approaches, with accuracy increases ranging from 1.72% to 11.25%.

Other research efforts have focused on analyzing different CNN architectures to compare their performance in facial emotion recognition. For instance, Chowdary et al. (2023), present a facial emotion recognition system utilizing transfer learning. The study employs pre-trained CNNs, including VGG-19, ResNet-50, Inception-v3, and MobileNet, with experiments conducted on the CK+ database. The results show accuracies of 96% for VGG-19, 97.7% for ResNet-50, 98.5% for Inception-v3, and 94.2% for MobileNet. Notably, MobileNet achieved the highest accuracy among the four networks, demonstrating its effectiveness in emotional facial recognition. Similarly, Sahoo et al. (2023) reports comparable results, emphasizing that the pre-trained VGG-19 model outperformed other models, such as AlexNet and SqueezeNet, on most benchmark databases. Compared to state-of-the-art technologies, the VGG-19 model achieved an accuracy of 99.7%. These results are considered a reference point for implementing transfer learning with the VGG-19 network to extract the probability vector in the present investigation.

Emotion recognition is currently used in various fields such as education, gaming, robotics, medical care and also in the automotive field, which is why new emotion models require more research to address the various challenges that exist around emotion recognition through facial expressions. The work of Bakariya et al. (2024), creates a real-time system that can analyze unstructured data capable of recognizing human faces, evaluate emotions and even make recommendations based on a deep learning approach. The accuracy of their proposal is 73.02%, objectively recognizing 6 emotions such as: anger, fear, joy, neutrality, sadness and surprise. In the same way Talaat et al. (2024), developed a real-time emotion identification system to detect emotions but in autistic children, using an autoencoder for feature extraction and selection, and applying transfer learning with different CNNs as a reference due to the reduced number of data. The Xception model achieved the highest performance with an accuracy of 95.23% demonstrating the ability of the procedure to recognize emotions. The study in question also establishes the feasibility of using transfer learning which is a critical point within the present research, well as Gursesli et al. (2024), which proposes a significant reduction of computational power and complexity for emotion recognition based on existing architectures such as MobileNetV2. Similarly, Ravikumar et al. (2024), used transfer learning and data augmentation procedures for model generalization using multiple reference data, concluding that a deep learning model based on transfer learning is recommended for recognizing emotions from facial expressions.

However, work such as Mehrotra et al. (2024), states that previous research focuses primarily on accuracy without taking into account prediction time which is also critical for an optimal emotion recognition system. Their suggested approach achieved an accuracy of 71.61% in a time of 58 minutes in the training process using the FER dataset.

### 2.2 Speech signals for emotion recognition

Some studies have proposed the development of Speech Emotion Recognition (SER) systems based on features extracted from spectrograms, implementing artificial neural network architectures. Mustaqeem et al. (2020) presents a significant method for selecting essential speech signal segments using a Radial Basis Function Network (RBFN). The selected segments are converted to spectrograms and passed to a CNN model to extract silent and discriminative features. These CNN features are normalized and fed into a deep bi-directional long short-term memory (BiLSTM) network for learning temporal features to recognize emotions. Similarly, Yao et al. (2020) developed an integrated framework combining Deep Neural Networks (DNN), CNN, and Recurrent Neural Networks (RNN). In their approach, the utterance-level outputs of high-level statistical functions (HSF), segment-level Mel-spectrograms (MS), and frame-level Low-Level Descriptors (LLDs) are inputted to DNN, CNN, and RNN, respectively. This yields three separate models–HSF-DNN, MS-CNN, and LLD-RNN. A multi-task learning strategy is employed across the models to extract generalized features by simultaneously performing emotional attribute regression and discrete emotion category classification.

Despite the rise of deep learning techniques, recent studies propose less computationally expensive methodologies in terms of time and performance. For example, Daqrouq et al. (2024) evaluates the performance of various machine learning algorithms, such as Support Vector Machine (SVM), K-Nearest Neighbors (KNN), Logistic Regression, Naive Bayes, and neural networks, by using discrete wavelet transform (DWT) with linear predictive coding (LPC). These findings can help guide the selection of appropriate classifiers and feature extraction methods for future research and real-world applications that use speech as a source of information.

The Mel Frequency Cepstral Coefficient (MFCC) method is widely employed for analyzing speech signals and has demonstrated superior performance in speech-based emotion recognition systems compared to other features. Alluhaidan et al. (2023) presents an emotion recognition model using hybrid features extracted from MFCC and the temporal domain, with a One-Dimensional Convolutional Neural Network (1D CNN). They use publicly available datasets such as EMO-DB, SAVEE, and RAVDESS to evaluate their method's performance, achieving precision rates of 96.6% for EMO-DB, 92.6% for SAVEE, and 91.4% for RAVDESS. The fusion of hybrid features with 1D CNN proved effective for speech emotion recognition, outperforming both conventional and deep learning approaches. Similarly, Bhangale and Kothandaraman (2023) explores emotion recognition using acoustic features and 1D CNN. Their study focuses on analyzing various acoustic features, such as MFCC, Linear Predictive Cepstral Coefficients (LPCC), Wavelet Packet Transform (WPT), Zero-Crossing Rate (ZCR), and Root Mean Square (RMS), to enhance the distinctiveness of speech signals. They develop a deep 1D CNN to reduce computational complexity in emotion recognition, testing its effectiveness on datasets like EMO-DB and RAVDESS. Their results demonstrate high accuracy for recognizing various emotions, including 94.83% accuracy for anger, 91.38% for calmness, 89.66% for disgust, 89.66% for fear, and 91.38% for happiness.

Recent studies continue to explore variations of MFCC. For instance, Mishra et al. (2024) extracted the MFCC coefficient matrix from various datasets, calculating features such as the statistical mean, MFCC-based approximate entropy, and MFCC-based spectral entropy. Their model achieved classification accuracies of 85.61%, 77.54%, and 76.26% across three different speech datasets.

There are also cases, such as in Khan et al. (2024), where it is suggested that reliable and robust multimodal speech emotion recognition systems are necessary to efficiently recognize emotions across multiple modalities, such as speech and text. In their proposal, a deep feature fusion technique for audio and text signals is applied to predict the emotion label. The proposed model processes raw speech and text signals using a CNN and employs encoders for semantic and discriminative feature extraction. The authors evaluate their model on various datasets and conduct extensive experiments, obtaining significant results that highlight the robustness and versatility of models trained on data from different sources.

However, the complexity of speech signal characteristics continues to present many challenges in emotion recognition. A study by Yang et al. (2024) introduces a multi-feature approach that aims to reduce the dimensionality of features to effectively address overfitting issues. Their experiment achieved remarkable accuracy on diverse datasets, with their model reaching accuracies of 98.47% and 98.87%, demonstrating the ability to accurately discern emotions from speakers. These findings underscore the importance of incorporating feature reduction in models that use speech cues as a primary source of information. This is an important consideration for the research presented in this manuscript, as reducing the dimensionality of motor cues will likely contribute to developing an optimal model for emotion recognition.

### 2.3 Biophysiological signals

Physiological signals are biochemical responses to stimuli that can be useful in identifying emotions. These data may include Electrocardiogram (ECG) signals, Electroencephalogram (EEG) signals, Electromyogram (EMG) signals, Galvanic Skin Response (GSR), and Heart Rate (HR). Methods based on biophysiological signals have shown promising results in emotion recognition. Recent studies, such as Yang et al. (2023), propose an LSTM system that combines smartphone sensors to capture images of the driver and a bracelet to record electrodermal activity, accurately determining the user's emotional state. The system was evaluated through a user study with 45 participants, using affective responses (facial expressions, speech, keystroke typing) and physiological responses (blood volume, electrodermal activity, and skin temperature) induced by visual stimuli.

Alternatively, other researchers have explored different methodologies for detecting emotions through ECG. For instance, Wu and Chang (2021) conducted experiments using ECG to investigate the impact of music on emotions. Their findings indicated that fast, intermediate, and slow music influenced the autonomic nervous system in different ways: fast music stimulated it, intermediate music inhibited it, and slow music had no significant effect. Additionally, they suggested that music could help alleviate psychological pressure. Hu and Li (2022) collected 140 ECG signal samples triggered by Self-Assessment Manikin (SAM) experiments using the International Affective Picture System. They employed a Wasserstein Generative Adversarial Network (WGAN) with a gradient penalty to augment different classes of samples. The results showed that increasing the quantity of data improved the accuracy and weighted F1 scores for all three classifiers.

Similarly, Fang et al. (2024) applied an emotion recognition method using random convolutional kernels for ECG signals. This approach reduces computational complexity and training time compared to methods that rely on multiple physiological signals or deep neural networks. It was validated on three publicly available datasets, achieving average recognition accuracies of 93.7%, 95.5%, and 91.5% in the valence, arousal, and dominance domains, following the three-dimensional approach to emotions proposed by Russell. Likewise, the study presented by Sweeney-Fanelli and Imtiaz (2024) implements deep learning techniques for emotion recognition using ECG signals, achieving accuracies of 98.68% for arousal and 97.30% for valence on two publicly available datasets. The results highlight the potential of temporal convolutional neural networks to enhance human-computer interactions and healthcare monitoring systems through improved emotion recognition. Additionally, Arslan et al. (2024) focuses on the processing and analysis of ECG and GSR signals to develop a predictive model for emotion classification. Their methodology involves extracting key features such as heart rate variability, morphological features, and Hjorth parameters. A feature selection process based on statistical analysis is applied to optimize and adapt the data for machine learning techniques, resulting in a classification accuracy of 97.78%. This demonstrates the feasibility of real-time emotion recognition through statistical feature selection and machine learning algorithms.

Changes in human emotions involve a complex process that often triggers spatiotemporal brain activity, which can be detected by EEG. EEG signals contain valuable information, as they reflect the activity of countless neurons in the cerebral cortex, providing real-time insights into brain functioning. Moreover, EEG recording is relatively simple and cost-effective, making EEG-based methods highly attractive for emotion recognition and evaluation.

Studies have shown a correlation between EEG signals and different emotional states. For example, Andreu-Perez et al. (2021) conducted a study in which participants played the video game "League of Legends" while their brain activity was monitored using functional near-infrared spectroscopy (fNIRS), along with video recordings of their facial expressions. They decoded the players' expertise level within a multimodal framework, achieving a tri-class classification precision of 91.44%. Similarly, Zhang J. et al. (2020) measured EEG signals, extracted features, and processed the data using a modified radial basis function neural network algorithm. Their experimental results demonstrated the superiority of this modified algorithm over others. In a related study, Zangeneh Soroush et al. (2020) reconstructed EEG phase space, extracted Poincaré intersections as features, and integrated them into a classification model, introducing an effective method for emotion recognition and nonlinear signal processing. Likewise, Lu et al. (2020) analyzed people's physiological responses to different lighting conditions based on EEG signals. Their findings revealed that illumination levels and color temperatures significantly impact the visual center's response, which can help design optimal lighting environments.

In recent times, EEG-based emotion recognition models continue to evolve. Cai et al. (2024) introduced a new EEG input format called EEG spectral imaging, which integrates spatial domain features using Azimuthal Equidistant Projection (AEP) and frequency domain features through differential entropy. Experiments performed on the SEED and SEED IV datasets demonstrated superior performance compared to benchmark methods and state-of-the-art models. Their results showed relative improvements of 0.6% and 0.08% in subject-dependent experiments, achieving accuracies of 80.07% and 66.72%, respectively. The author also notes that existing approaches (Trujillo et al., 2024; Al-Asadi et al., 2024; Tokmak et al., 2024; Jha et al., 2024) mainly focus on the time and frequency domain characteristics of EEG signals.

Unlike behavioral data, physiological data are considered more objective because they typically reflect involuntary responses that are difficult to consciously conceal or alter (Lin and Li, 2023). However, much of the research in this field depends on medical-grade physiological sensing equipment, which tends to be invasive, expensive, and requires technical expertise, thus limiting its application in real-world settings (Dunn et al., 2018).

In the study by Siam et al. (2023), an approach is proposed to identify the mental stress of automotive drivers based on selected biosignals such as ECG, EMG, GSR, and respiration rate. Six different machine learning models were employed to classify stress and relaxation states. The proposed Stress Detection Technique (SDT) consists of three main phases: biosignal preprocessing, feature extraction, and classification. The results show that Random Forest outperformed other techniques, achieving a classification accuracy of 98.2%, sensitivity of 97%, and specificity of 100% using a public driving dataset. This research aims to integrate biosignals with the automotive industry to develop an applicable Advanced Driver Assistance System (ADAS). Additionally, Waheed Awan et al. (2024) suggests a method based on a one-dimensional convolutional neural network and Vision Transformer, where the process involves decomposing signals into segments, removing noise, and extracting features. These features are then integrated into a single vector for classification using a set of classifiers. The results are synthesized using Model Agnostic Meta Learning (MAML) to improve prediction accuracy. The model was validated on the AMIGOS and DEAP datasets, achieving up to 98.2% accuracy with 10-fold cross-validation, leveraging physiological signals for comprehensive emotion assessment.

### 2.4 Emotion recognition in automotive field

Although it is challenging to present a definitive or statistical figure for accidents caused by drivers' emotions, some researchers have worked on proposals to determine drivers' emotional states in real time, based on the analysis and processing of various sources of information, in order to prevent accidents in advance. The following are studies related to emotion recognition in an automotive environment.

Different methods for objectively determining drivers' emotions have been explored. For example, Wang et al. (2020) used feature fusion of multiple ECGs to detect driver emotions based on a backpropagation network and the Dempster-Shafer evidence method. In their approach, they selected ECG signals, time-frequency domain, waveform, and nonlinear features as parameters for emotion recognition, specifically identifying drivers' calmness and anxiety while driving. The results demonstrated that after fusing the ECG parameters, the proposed model could recognize drivers' emotions, with an accuracy of 91.34% for calmness and 92.89% for anxiety. The study concludes that this method holds theoretical and practical significance for improving road safety.

Other proposals present new multimodal frameworks for emotion recognition, integrating facial expressions and heart rate data. For instance, Du et al. (2021) established a deep learning model called the Bidirectional Convolutional Long-term Memory Neural Network (CBLNN). This model predicts drivers' emotions based on geometric features extracted from changes in RGB components. The facial features obtained using the CNN serve as intermediate variables for Bidirectional LSTM (BI-LSTM) heart rate analysis. The BI-LSTM output is then used as input to the CNN module to extract features. CBLNN applies multimodal factorized bilinear clustering to fuse the extracted information and classify five common emotions. This emotion detection method achieved recognition rates of 91.6%, 90.50%, 91.51%, and 89.15% for happiness, anger, sadness, and neutrality, respectively.

The implementation of artificial intelligence algorithms has been a critical tool for objectively recognizing emotions in drivers, due to their capacity to extract features that identify the emotions we experience throughout the day. Naqvi et al. (2020) based their method on gaze changes and facial emotions using NIR camera sensors and an illuminator installed in the vehicle. They acquired time-series data from aggressive and normal drivers by simulating driving scenarios with racing and truck driving games. Software was used to capture the driver's image data, extracting images of the face and eyes to detect gaze changes with a CNN and classify facial emotions. Score-level fusion was applied to the scores obtained from gaze changes and facial emotions to classify aggressive and normal driving. The accuracy of their method, measured through a self-generated test database, achieved a classification accuracy of 98.93%. Similarly, Cui et al. (2020) implemented CNN for emotion recognition, where a multitask network processed facial expressions under varying lighting conditions. This was accomplished by simultaneously restoring corrupted images using a pre-trained CNN and predicting specific emotions. Their model, evaluated on the FerPlus and Cohn-Kanade (CK+) datasets (Lucey et al., 2010), achieved an accuracy of 83.1%.

Recent studies continue to advance emotion recognition techniques, with a focus on image analysis and processing. For example, Xiao et al. (2022) proposed a method that includes three modules: facial detection, a data resampling module, and an emotion recognition module based on a deep CNN pre-trained with FER (Barsoum et al., 2016) and CK+ datasets. This method, designed for real-time emotion recognition in drivers, collected on-road facial expression data in various driving scenarios, achieving an emotion recognition performance of 97.2%. Similarly, Zaman et al. (2023) used CNN, RNN, and multilayer perceptron classification models to develop a facial expression recognition system. Their model, built on the faster region-based enhanced CNN (R-CNN) for real-time face detection, fused CNN model features to train an emotion classification model. By incorporating InceptionV3 into the model, they improved accuracy, achieving recognition rates of 98.01%, 99.53%, 99.27%, 96.81%, and 99.90% across several datasets, including JAFFE (Lee and Kang, 2020), CK+, FER2013 (Zahara et al., 2020), AffectNet (Mollahosseini et al., 2017), and their own dataset. Additionally, Sukhavasi et al. (2022) proposed a hybrid methodology combining CNN and Support Vector Machine (SVM) to enhance classification predictions. By fusing Local Binary Patterns (LBP) and Gabor filters to extract robust features, their technique achieved accuracies of 84.41%, 95.05%, 98.57%, and 98.64% on FER-2013, CK+, KDEF (Goeleven et al., 2008), and KMU-FED (Kumar et al., 2022), respectively.

Another innovative study by Jain et al. (2023) proposed the development of an algorithm called Squirrel Search Optimization with Deep Learning Enabled Facial Emotion Recognition (SSO-DLFER) for detecting emotions in autonomous vehicle drivers. This algorithm employed the RetinaNet (Lin et al., 2017) for face detection and the NASNet-Large (Zoph et al., 2018) feature extractor with the Gated Recurrent Unit (GRU) classifier for emotion recognition. Hyperparameter tuning based on SSO enhanced the model's performance, achieving a maximum accuracy of 99.50% across multiple datasets, including KDEF and KMU-FED.

A notable study that deviates from image analysis is presented by Chen et al. (2024), who explored the relationship between EEG signals and emotions in a simulated driving environment. Their method used vehicle speed as a variable to simulate obstacle avoidance at different danger levels. For data processing, graphical neural networks with functional connectivity and attention mechanisms were employed to simulate the brain's physiological structure. Their binary classification result achieved an F1 score of 91.5%, demonstrating the effectiveness of capturing EEG signals and monitoring emotional states through deep learning models.

In another study, Mou et al. (2023) introduced a multimodal fusion framework for driver emotion recognition, employing a ConvLSTM network with a hybrid attention mechanism to integrate eye, vehicle, and environmental data. Their research revealed correlations between driver emotions and stress, with participants exhibiting higher levels of valence and emotional dominance under stressful conditions. The model achieved average precision values of 97.64% for valence, 97.27% for arousal, and 96.47% for dominance, further validated through ablation experiments.

Several studies have examined drivers' behavioral characteristics, such as motor activity signals influenced by emotional states. For instance, CAN bus signals are commonly used, though access to these signals is restricted to in-house developers (Zepf et al., 2020). Despite the growing interest in driver behavior, many studies lack sufficient detail about the characteristics and behaviors associated with different emotions. One of the least explored methodologies in a driving context is the use of multimodal artificial intelligence (AI) models. These models, which process data from various sources, have shown potential in emotion recognition. Oh et al. (2021) proposed an emotion recognition model that fused facial expression data with electrodermal activity, achieving an accuracy of 86.8%. Likewise, Zhou et al. (2023) proposed a multimodal model integrating driver voice, facial images, and video sequences using CNN, Bi-LSTM, and hybrid attention modules, recognizing six negative emotions (e.g., sadness, anger, fatigue) with an accuracy of 85.52%. Ying et al. (2024) similarly employed audio and video features to recognize driver emotions, enhancing the safety and humanization of advanced driver-assistance systems (ADAS).

The current state of the art highlights the feasibility of developing reliable emotion recognition systems suitable for real-world implementation. However, the exploration of multimodal AI models that integrate behavioral and facial data remains largely unexplored. Mou et al. (2023) have shown potential in emotion identification tasks within automotive environments. Such advancements can improve the driving experience and enhance road safety by mitigating aggressive or distracted behaviors, ultimately benefiting society.

Exploring alternatives for accurate, efficient emotion recognition remains complex, yet research on behavioral signals continues to provide insights. For instance, Paredes et al. (2018) demonstrated the ability to measure driver strain using a steering angle and a mass-spring-damper model. Other studies have established correlations between emotional states and driving behaviors. Hu et al. (2024) proposed a multimodal emotion recognition model using facial videos and driving behavior (e.g., brake pedal force, Y-axis position, vehicle Z-axis position), achieving an accuracy of 63.83% in a first approach to intelligent emotion recognition based on facial and behavioral features.

In conclusion, while significant progress has been made, challenges remain in improving the accuracy and robustness of emotion recognition in dynamic environments. Integrating facial data with motor activity data, such as steering wheel and pedal interactions, could provide a richer context for recognizing drivers' emotions, enhancing both the accuracy and practicality of emotion recognition systems for real-world applications.

## 3 Materials and methods

**Figure 1:**
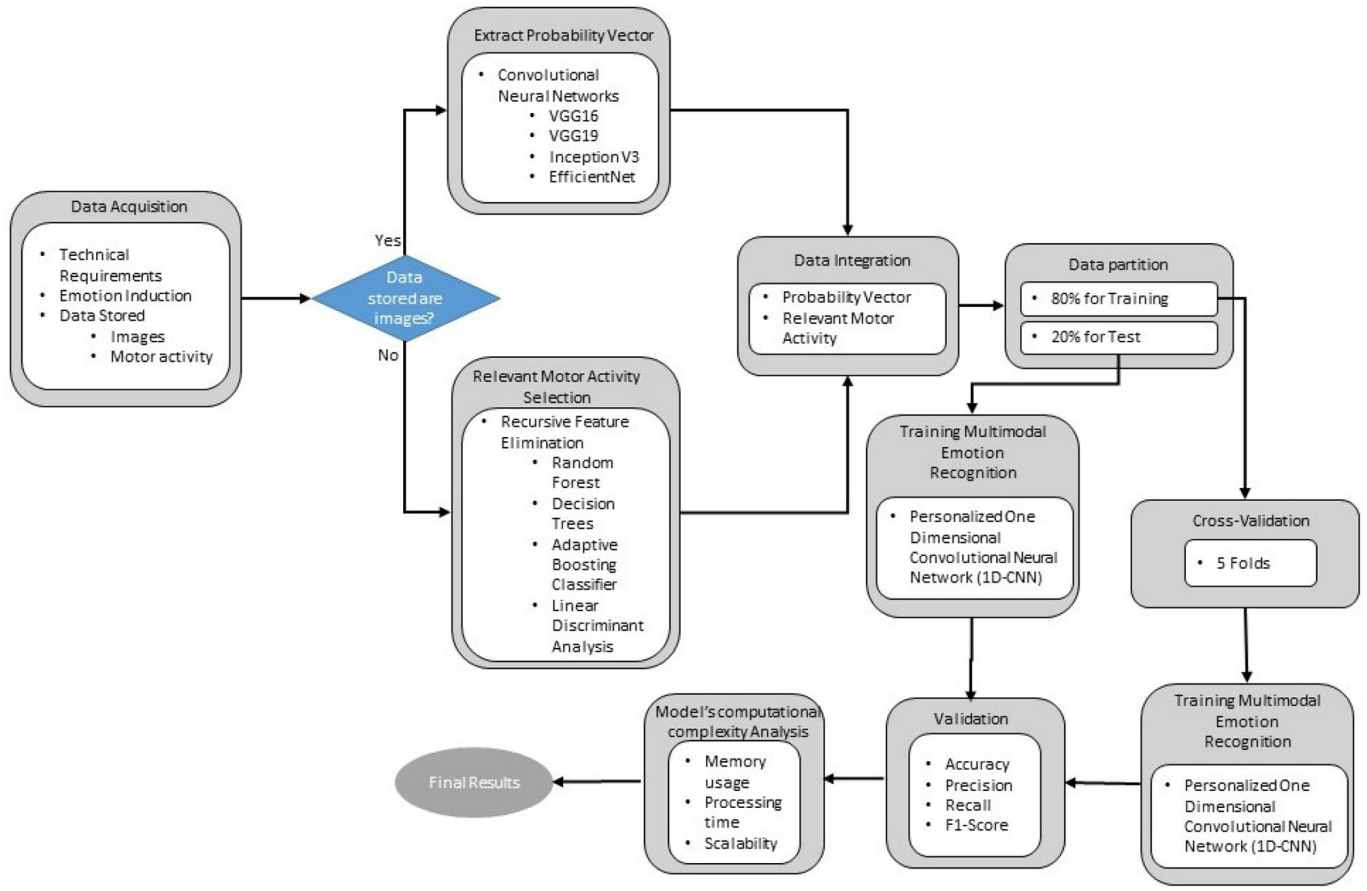
Proposed methodology for generating a dataset of motor activity and facial images of drivers under different induced emotional states, and a multimodal emotion recognition model using convolutional neural networks.

Figure 1 presents the methodology followed in this research to develop a multimodal emotion recognition model. The model primarily utilizes the motor activity or behavior of drivers and geometric patterns of facial expressions as inputs. The first stage, data acquisition, focuses on generating a dataset of motor activity signals obtained from key vehicle elements, such as steering wheel angle, pedal movement, and braking, alongside visual data like facial images of the participants. These data are collected during the induction of four emotions in a simulated driving environment, using emotion neutralization and the augmented autobiographical recall technique.

In the second stage, the probability vector of images for each induced emotion is extracted using a pre-trained Convolutional Neural Network (CNN). The third stage involves selecting the most relevant driving signals for emotion recognition by applying intelligent feature selection methods with machine learning algorithms such as Random Forest Classifier (RFC), Decision Trees (DT), Adaptive Boosting Classifier (ABC), and Linear Discriminant Analysis (LDA). The fourth stage processes both motor signals and probability vectors through a One-Dimensional Convolutional Neural Network (1D-CNN) to generate a multimodal model capable of recognizing a limited set of emotions. This step marks a preliminary attempt at analyzing and processing these types of data using advanced artificial intelligence algorithms simultaneously.

In the fifth and final stage, the model is validated using key performance metrics from the field of artificial intelligence to evaluate its accuracy in identifying emotions in a driving environment. Additionally, a computational complexity analysis is performed to determine the model's viability for real-time inference in automotive systems.

### 3.1 Data acquisition

**Figure 2:**
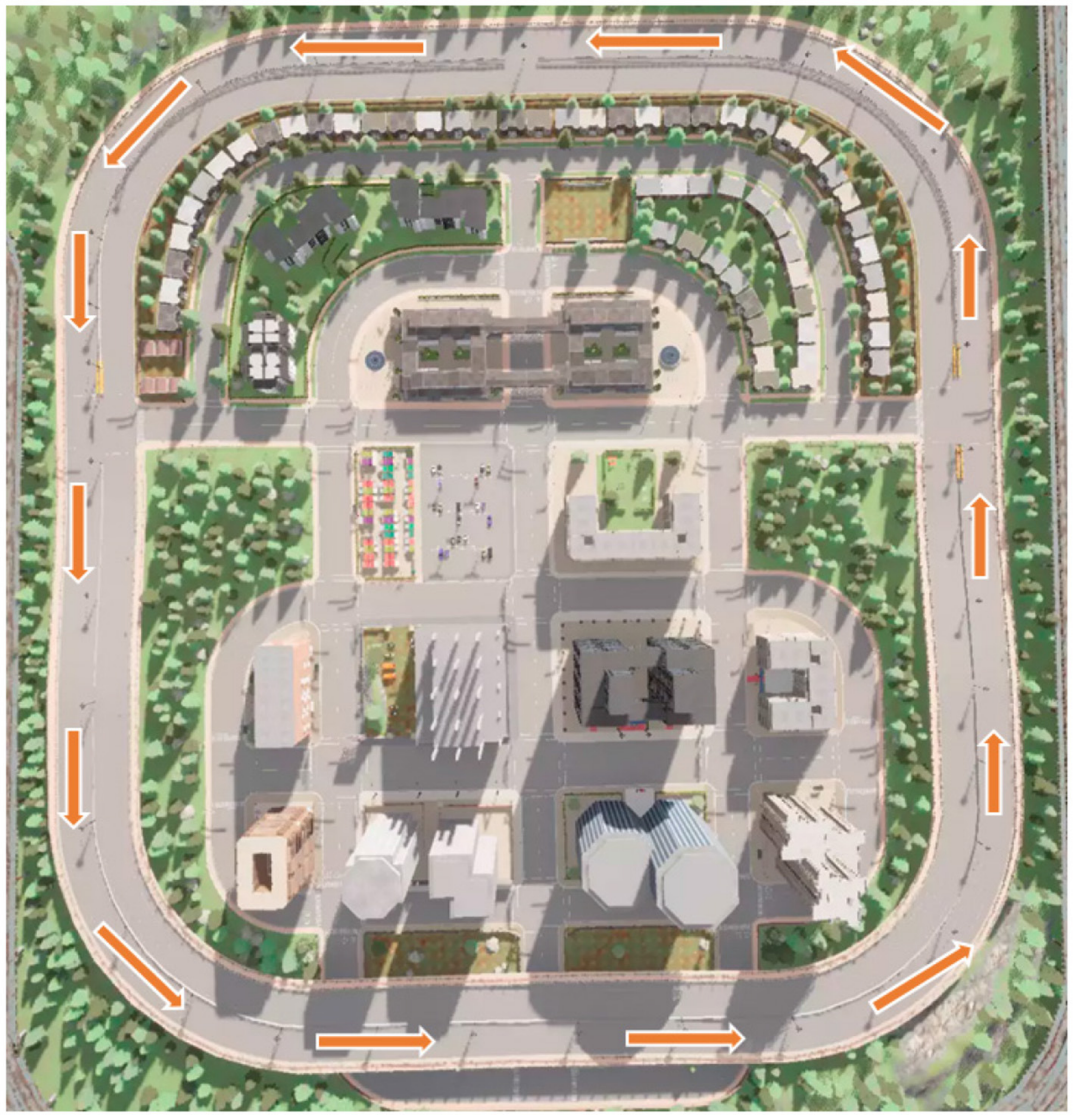
Map of the CARLA simulator used for driving simulation and the established route. The orange lines represent the route followed by each of the participants.

The experimental tests were conducted using the open-source driving simulator CARLA 0.9.13 for safety reasons. CARLA was developed to support the creation, training, and validation of autonomous vehicles, and it is widely used for advanced driver assistance system research, including algorithm training for perception tasks. CARLA is freely available, and its sensor configuration settings allow for the collection of signals that can be used to train driving strategies (Dosovitskiy et al., 2017). For this research, the same scenario as shown in Figure 2 (whose specifications can be found here: https://carla.readthedocs.io/en/latest/map_town05/) was used for all participants and emotions tested. Each participant followed a pre-established route under uniform virtual conditions, adhering to real-world traffic rules. As the tests were conducted in a controlled driving environment, only active driving data were collected. This included recording the steering wheel angle (-180 to 180 degrees), brake pedal movement (-1 to 1), and throttle pedal movement (-1 to 1), as driver behavior can be influenced by emotional state, especially in interactions with the vehicle such as steering adjustments and pedal usage (Zepf et al., 2020).

To capture motor activity data, the Logitech Driving Force G29, which includes the steering wheel, throttle pedal, and brake pedal, was used. These peripherals are designed specifically for driving simulations, making them ideal for collecting essential data. The simulator's built-in properties were leveraged to record critical information, including the steering wheel angle and the amount of movement in both the brake and throttle pedals.

For capturing images of the region of interest (ROI)—in this case, the driver's facial geometry—a LOGITECH C270 camera with 720 megapixels was used.

The computer used for the experiment was equipped with an Intel Core i5-9400F processor running at 2.90 GHz, 32 GB of RAM, and an NVIDIA GeForce GTX 1070 Ti graphics card.

Each participant signed an informed consent form, following the ethical guidelines established in the Helsinki Declaration.

Various methods exist for inducing emotions, but augmented autobiographical recall has proven particularly effective in driving environments, as suggested by Braun et al. (2018). This method is advantageous because it allows the participant to generate the emotional stimulus themselves, reducing the risk of misinterpretation. Additionally, it can be smoothly integrated into driving tasks, offering a seamless transition from emotional provocation to the driving experience.

In this method, participants are asked to recall and write about a past event to evoke a specific emotion. They are encouraged to provide as many details as possible and vividly recount the events. Crucially, participants recall the story to themselves, without the experimenter's presence (López-Cano et al., 2020). To further enhance the emotional experience, scientifically validated songs known to evoke specific emotions were played through headphones. These auditory stimuli, sourced from the DEAP dataset (Koelstra et al., 2012), intensified the emotions participants experienced during the drive. Table 1 lists the songs used in the experiment.

**Table 1:** Song titles, artist who performs it and the type of emotion it can evoke, obtained from the DEAP data set.

**Emotion**	**Artist**	**Title**
Happiness	M. Franti and spearhead	Say Hey (I Love You)
Sadness	James Blunt	Goodbye my lover
Anger	Dead To fall	Bastard set of dreams

Before beginning the augmented autobiographical recall, participants evaluated their emotional state using an affective annotation platform that featured the Self-Assessment Manikin (SAM). SAM is a widely used tool for assessing emotional states, featuring a graphic scale for valence and arousal from 1 to 9 (Veeranki et al., 2021). After this initial evaluation, participants were asked to write about a neutral event, such as their morning toothbrushing routine, to establish a neutral emotional state. This step follows the methodology of Sanghavi et al. (2020), which demonstrates that recalling a mundane event effectively induces neutrality. Once participants had completed this task, they put on headphones and began the driving simulation, during which they listened to a song designed to maintain a neutral emotional state. This initial phase not only familiarized participants with the simulator but also prepared them for the emotional recall process.

After a 5-minute neutral driving session, participants reassessed their emotional state on the platform. They were then asked to recall a moment that elicited one of the target emotions for the study (Happiness, Anger, or Sadness) and repeated the same tasks as in the neutral driving phase. Finally, participants evaluated their emotional state again, using the augmented autobiographical recall method during driving.

**Figure 3:**
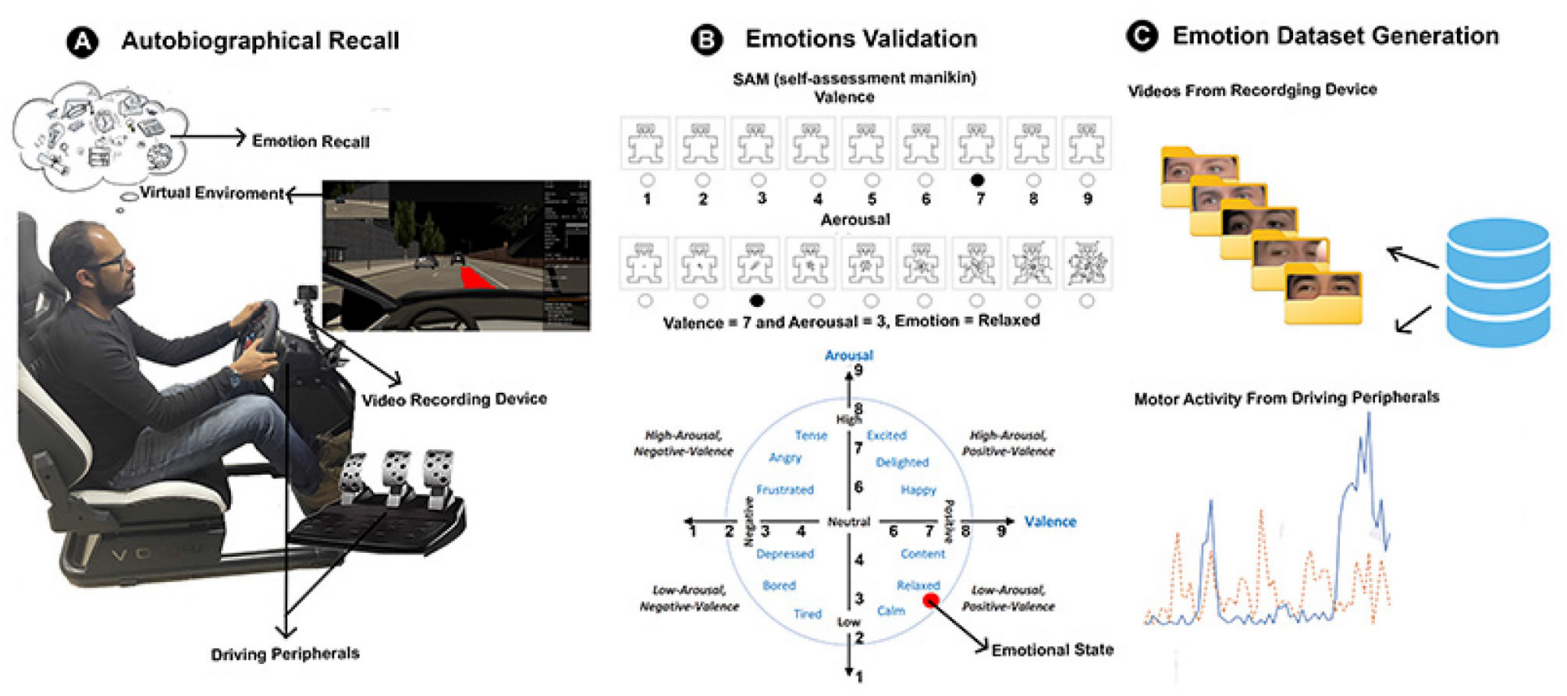
Proposed methodology for generating a dataset of motor activity and facial images of drivers under different induced emotional states, and a multimodal emotion recognition model using convolutional neural networks.

All collected data, including motor activity and facial images, were organized by the emotion associated with each participant, ensuring control over records and allowing for efficient storage. This organization adhered to the emotion categories of Happiness, Anger, and Sadness, selected based on Plutchik's emotional model. According to Plutchik, these basic emotions serve as building blocks for more complex emotions (Semeraro et al., 2021). Additionally, focusing on a smaller set of emotions allowed for a more representative and high-quality dataset, ensuring that the data were well-labeled and balanced (Khoo et al., 2024). Figure 3 summarizes the data acquisition process.

### 3.2 Probability vector extraction

In this study, pretrained Convolutional Neural Network (CNN) models, including VGG16, VGG19, Inception V3, and EfficientNet, were employed. These models are widely used and straightforward, and recent studies (Zaman et al., 2023; Gite et al., 2023; Tauqeer et al., 2022; Oh et al., 2021; Ahmad et al., 2024; Wawage and Deshpande, 2022) have demonstrated their remarkable performance across a variety of applications, including emotion recognition. The objective of the pretrained CNNs is to calculate the probability vector of facial geometry images for each induced emotion obtained from participants during simulated driving using transfer learning (Kusal et al., 2024).

The probability vector represents the distribution of probabilities across different emotions, which helps reduce the dimensionality of the data compared to raw image feature extraction. Emotion recognition often involves addressing variability in facial expressions, lighting conditions, and other environmental factors. The probability vector captures the uncertainty associated with these variations, making the model more robust to changes in the input data. By focusing on the probability distribution of emotions, rather than specific image features, the model achieves a more nuanced understanding of the underlying semantics of facial expressions. In some cases, using a probability vector enables end-to-end learning, allowing the model to directly map input images to probability distributions of emotions (Zhao et al., 2020).

**Figure 4:**
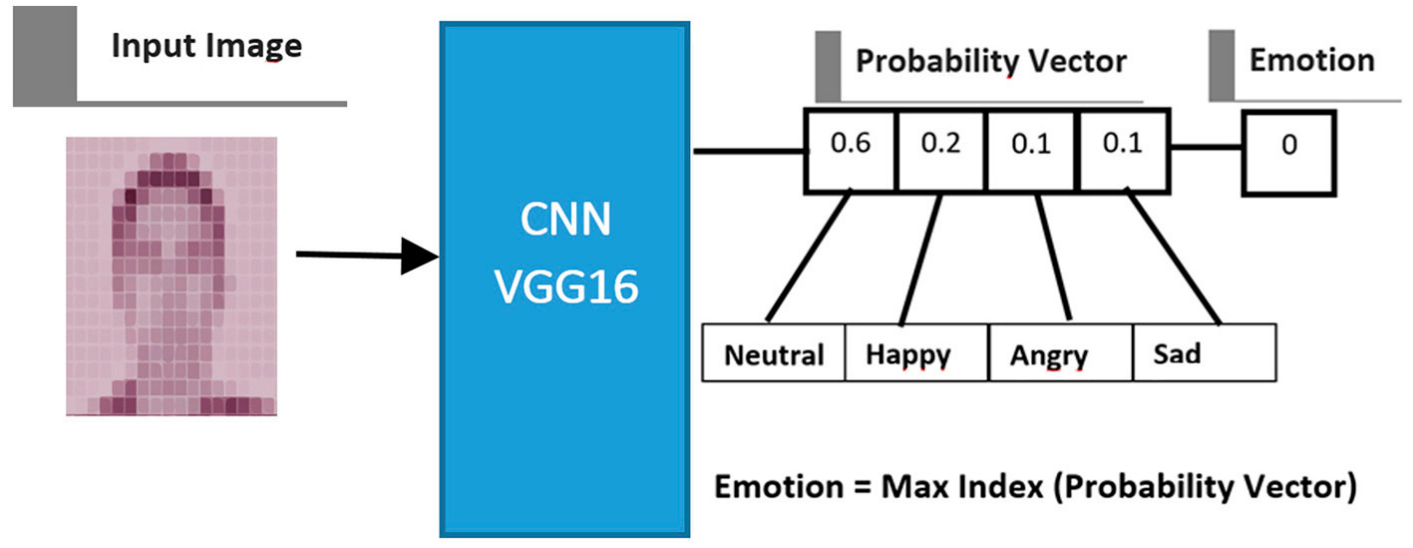
Process of extracting probability vector from an image using a CNN.

The probability vector is generated before assigning a label to each input image. The number of elements in the probability vector corresponds to the number of induced emotions in the data acquisition process (Neutral, Happy, Angry, and Sad), with each element representing the likelihood of one specific emotion. Since the total probability is 1, the sum of all elements in the probability vector equals 1, and each element's value ranges between 0 and 1. The emotion associated with the highest value in the probability vector is selected as the detected emotion. Figure 4 illustrates the probability vector extraction process. These probability vectors, derived from the facial images, are a key complement to the tabular dataset (e.g., throttle pedal movement, brake pedal movement, and steering wheel angle) in the integration phase.

The VGG16 CNN was developed by the Visual Geometry Group (VGG) at the University of Oxford and became well-known after winning the ImageNet Large Scale Visual Recognition Challenge (ILSVRC) in the object identification category. This model has also shown very promising results in emotion recognition in other studies (Verma and Choudhary, 2018a,b). The goal of this network is to demonstrate that increasing the depth of the network can improve performance in certain tasks (Shahzad et al., 2023). The VGG19 network, proposed by Simonyan and Zisserman, consists of 19 layers, including 16 convolutional layers and 3 fully connected layers, and it is trained to classify 1000 different objects. VGG19 is trained on the ImageNet database, which contains over one million images (Bansal et al., 2023).

In contrast, the Inception V3 network is a deep architecture developed for the 2014 ImageNet visual recognition challenge. One of its main advantages over VGGNet is its faster execution speed (Cao et al., 2021). Finally, EfficientNet is another pretrained CNN, designed for transfer learning in image classification tasks. This architecture, developed by Google AI in May 2019, is available through TensorFlow and GitHub libraries (Marques et al., 2020).

### 3.3 Feature selection

Once the tabular data from the driving simulator peripherals, including throttle movement, braking, and steering wheel angle, are collected for the different induced emotional states of the participants, a feature selection process is performed using the Recursive Feature Elimination (RFE) technique. This is necessary due to the high dimensionality of the motor activity data and the probability vectors derived from the images.

RFE is a commonly used feature selection technique in machine learning. The main idea behind RFE is to iteratively train a model on subsets of features and eliminate the least important ones at each iteration until the desired number of features is reached. The general algorithm is as follows (Ba et al., 2023):

- Initialization:
  Let *F* represent the set of all features, initially *F* = {1, 2, .., *p*} where *p* is the total number of features
- Iteration:
  For each iteration *i* (where *i* = 1, 2, ..):
  Train the model *M* using the features *X*_*F*_ and target variable *y*.
  Asses the importance of each feature based on some criterion, denoted as *I*(*f*) for feature *f* in *F*.
  identify the least important feature, *f*_*min*_ = *argmin*_*fϵF*_*I*(*f*). Remove the least important feature from *F*:*F*←*F*\{*f*_*min*_}.
- Stopping Criteria:
  Repeat the iteration until the desired number of features is reached or a predefined stopping criterion is satisfied.

The motor activity data vector consists of windows of 50 data points per record for steering wheel angle, throttle pedal movement, and braking movement. These data points are processed using RFE in conjunction with various machine learning techniques to identify the optimal data windows for emotion recognition based on variations in specific data segments collected under different emotional states. Ultimately, the most relevant data segments will be integrated with the probability vectors derived from the images. The machine learning techniques implemented for analysis and processing are described below.

#### 3.3.1 Random forest classifier

RFC is a machine learning algorithm that builds multiple decision trees, where each tree is generated using a random subset of the data. Each tree casts a vote, and the most popular class is selected to classify the input vector (Amiri et al., 2024).

RFC uses the Gini index as a measure for selecting attributes, which quantifies the impurity of an attribute with respect to the target classes. The Gini index is shown in Equation 1.


(1) $$\begin{array}{l} \sum \underset{j\neq i}{\sum }\left(f\left(C_{i},T\right)/|T|\right)\left(f\left(C_{j},T\right)/|T|\right) \end{array}$$

where *f*(*C*_*i*_, *T*) represents the frequency of class *C*_*i*_ in dataset *T*, and |*T*| is the total number of instances in *T*.

#### 3.3.2 Decision trees

Decision trees are supervised predictive models known for their interpretability and robustness, and they are widely applied in various domains. The fundamental idea behind a decision tree is to recursively divide the dataset into smaller subsets based on specific features until a strong prediction for the target variable is achieved. Each division is made in such a way that it maximizes the homogeneity of the resulting subsets in terms of the target variable (Costa and Pedreira, 2023).

#### 3.3.3 AdaBoost classifier

The goal of the AdaBoost algorithm is to combine multiple weak learners to form a strong learner, thereby improving the classification or prediction model. The algorithm works by adjusting the weights of the misclassified points at each iteration, giving more weight to incorrectly classified samples. A weak learner is trained using these weighted data points. A coefficient is assigned to each weak learner based on its performance. For misclassified points, their weights are increased, and the weights of correctly classified points are decreased. The process is repeated until all data points are correctly classified or a stopping criterion is met.

The AdaBoost algorithm is commonly used for binary classification problems but can be extended to handle multiclass classification using methods such as One-vs-All (OvA) or One-vs-One (OvO). The equation for the combined classifier *H*(**x**) is presented below:


$$\begin{array}{l} H\left(\text{x}\right)=\arg\underset{k}{\max}\overset{T}{\underset{t=1}{\sum }}\alpha_{t}\cdot I\left(h_{t}\left(\text{x}\right)=k\right) \end{array}$$

where *T* is the number of iterations, α_*t*_ is the weight of the *t*-th weak classifier, *h*_*t*_(**x**) is the prediction of the weak classifier, and *I* is the indicator function.

#### 3.3.4 Linear discriminant analysis

LDA is a supervised dimensionality reduction technique that aims to find a linear combination of features that maximizes the between-class variance while minimizing the within-class variance. In the transformed space, samples of the same class are separated as much as possible. For multiclass problems, LDA can be extended using Fisher's discriminant analysis to find a subspace that captures the maximum variability between classes (Zhu et al., 2022).

Suppose that each class *C* has a mean μ_*i*_ and a shared covariance matrix Σ. The between-class scatter matrix Σ_*b*_ can be defined as the covariance of the class means:


(2) $$\begin{array}{l} \Sigma_{b}=\frac{1}{C}\overset{C}{\underset{i=1}{\sum }}\left(\mu_{i}-\mu \right){\left(\mu_{i}-\mu \right)}^{T} \end{array}$$

where μ is the mean of the class means.

### 3.4 Model generation

Once the data integration process was completed, and the most significant motor signals were identified using machine learning algorithms, dimensionality reduction was performed using the Recursive Feature Elimination (RFE) technique. The resulting dataset comprised 3,361 observations and 13 columns, with the first 9 columns representing motor activity data and the last 4 columns representing the extracted probability vectors. This adjustment ensured consistency between the significant motor activity data used for emotion recognition and the number of extracted probability vectors, given that the number of images was much smaller than the motor activity dataset.

With the final dataset established, a multimodal emotion recognition model was developed using a proposed one-dimensional convolutional neural network (1D-CNN). 1D-CNNs are commonly utilized to analyze one-dimensional signals, such as vectors, time series data, and other sequential data types, and have been applied in various fields, including bioengineering, physiological signal analysis, traffic analysis, marketing, and network analysis (Tang et al., 2020).

The proposed network consists of five convolutional layers with filter sizes of 64, 128, 256, 512, and 1024, each using the Softplus activation function and kernel sizes of 3, 3, 2, 2, and 1, respectively. Softplus, known for its smoothness and non-zero gradient, was introduced by Dugas et al. (2000) in 2001 and, is defined as follows:


(3) $$\begin{array}{l} \text{Softplus}\left(x\right)=\ln\left(1+e^{x}\right) \end{array}$$

Additionally, max-pooling is applied at the end of the convolutional layers using a 3x3 kernel size. Four dense layers are then added with sizes 512, 256, 128, and 64, each with a Softplus activation function. The final layer, consisting of 4 units, uses a softmax activation function to predict the emotions.

The loss function used is Sparse Categorical Cross Entropy (SCCE), commonly employed in classification tasks where the target labels (*y*_true_) are provided as integers (class indices) instead of one-hot encoded vectors. SCCE simplifies the process when handling integer labels, though its formula is similar to that of categorical crossentropy (Chaithanya et al., 2021).

Equation 5 presents the mathematical representation of sparse categorical crossentropy:


(4) $$SCCE=-\frac{1}{N}\sum_{i=1}^{N}\sum_{j=1}^{C}y_{ij}\cdot \log({\hat{y}}_{ij})$$

Where:

- *N* is the number of samples.
- *C* is the number of classes.
- *y*_*ij*_ is a binary indicator of whether class *j* is the true class for sample *i*.
- ŷ_*ij*_ is the predicted probability that sample *i* belongs to class *j*.

**Figure 5:**
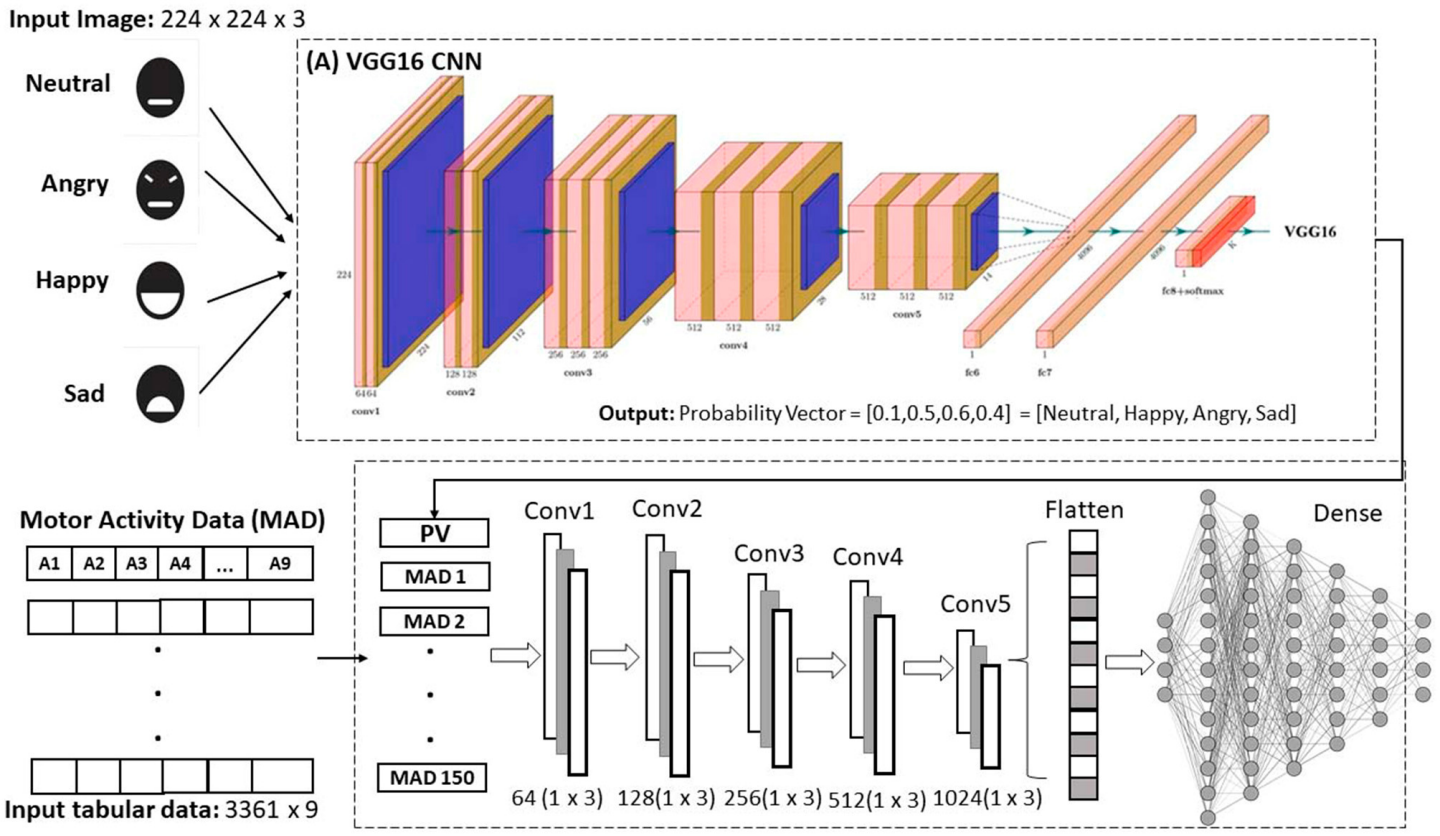
Proposed scheme for emotion recognition by fusing the image probability vector using the pre-trained CNN and tabular motor activity data using a one-dimensional convolutional neural network.

In summary, our approach integrates two types of CNNs: a two-dimensional network that extracts the probability vector from the visual dataset, and a one-dimensional network that processes motor activity signals in conjunction with the probability vector. This integration allows the model to process information from multiple sources and accurately and objectively identify emotions in drivers. The proposed architecture is summarized in Figure 5.

This data integration process has been scientifically validated in the study titled *Detection of Pedestrians in Reverse Camera Using Multimodal Convolutional Neural Networks* conducted by Reveles-Gómez et al. (2023).

### 3.5 Validation

To validate the performance of the model used in this study, a comprehensive set of evaluation metrics was employed. These metrics quantify the model's performance and assess its ability to effectively distinguish between different emotions. Each metric provides insights into various aspects of the model's quality, offering a holistic understanding of its behavior in classifying the emotions defined in this research.

The evaluation criteria applied include a range of metrics to ensure a thorough assessment of the model's efficacy. Among the metrics used are accuracy, recall, F1-score, and K-fold cross-validation. Together, these metrics provide valuable information about the model's classification performance, its ability to correctly identify each emotion category, and its robustness when evaluated through different validation techniques.

Accuracy is a fundamental metric that indicates the proportion of correctly classified instances among the total number of instances evaluated. Recall, on the other hand, measures the model's ability to correctly identify instances of a specific emotion class from all instances that truly belong to that class. The F1-score takes both precision and recall into account, providing a balanced assessment of the model's performance, particularly useful in cases where class distributions are imbalanced.

In addition to these metrics, K-fold cross-validation was used to evaluate the model's generalization capabilities and consistency across different subsets of the dataset. This technique involves dividing the dataset into *K* equally sized folds, training the model on *K*−1 folds, and then evaluating its performance on the remaining fold. This process is repeated *K* times, with each fold serving as the validation set once, ensuring the model's performance is not overly reliant on any single subset of the data.

By employing this comprehensive suite of evaluation metrics, we gain a deeper understanding of the model's strengths and weaknesses, ensuring a rigorous assessment of its performance in emotion classification tasks.

## 4 Results

For data acquisition, 50 participants (comprising 10 females and 40 males) aged between 18 and 39 years (with an average age of 25.26, a standard deviation of 5.36, and a median of 25.5) were recruited from the Autonomous University of Zacatecas (UAZ). These participants underwent 55 simulated driving tests at the Interactive Technologies and User Experience Laboratory (L.I.T.U.X) and had a minimum of one year of driving experience (with an average of 6.13 years, a standard deviation of 5.20, and a median of 5 years).

Each of the 50 participants selected for the experiment underwent an initial process of emotion neutralization before inducing the emotions established in this study, using the method of augmented autobiographical recall. Inducing emotions through imagination and music is particularly suitable for measuring direct effects, such as anger caused by aggressive driving, as established in the work of Steinhauser et al. (2018).

Each participant recalled and wrote down an event that produced a certain emotional state, which they later recalled during the test. Below is an example of a happy autobiographical memory:

“*When my son was born, holding him and feeling him in my arms was something incredibly special. Every day he hugs me and tells me he loves me, which makes me very happy.”*

The effectiveness of this methodology was assessed using the SAM (Self-Assessment Manikin) test, which measures emotional states across two dimensions: valence and arousal. During the driving tests, participants continuously characterized their emotional states using the SAM test. The Induced Emotion (IE) for each participant needed to closely align with the one characterized in the continuous model. As noted by Oh et al. (2021), if the induced emotion did not match the assigned values within the emotional range of the continuous model, the participant's data were discarded. Additionally, following the approach proposed by Li et al. (2022), the data were normalized using the min-max normalization method, as shown in Equation 5, to ensure uniform treatment of each arousal-valence value.


(5) $$\begin{array}{l} X_{norm}=\frac{X-X_{min}}{X_{max}-X_{min}} \end{array}$$

- *X*_*norm*_ represents the normalized values.
- *X* is the original value.
- *X*_*min*_ is the minimum value in the dataset.
- *X*_*max*_ is the maximum value in the dataset.

**Figure 6:**
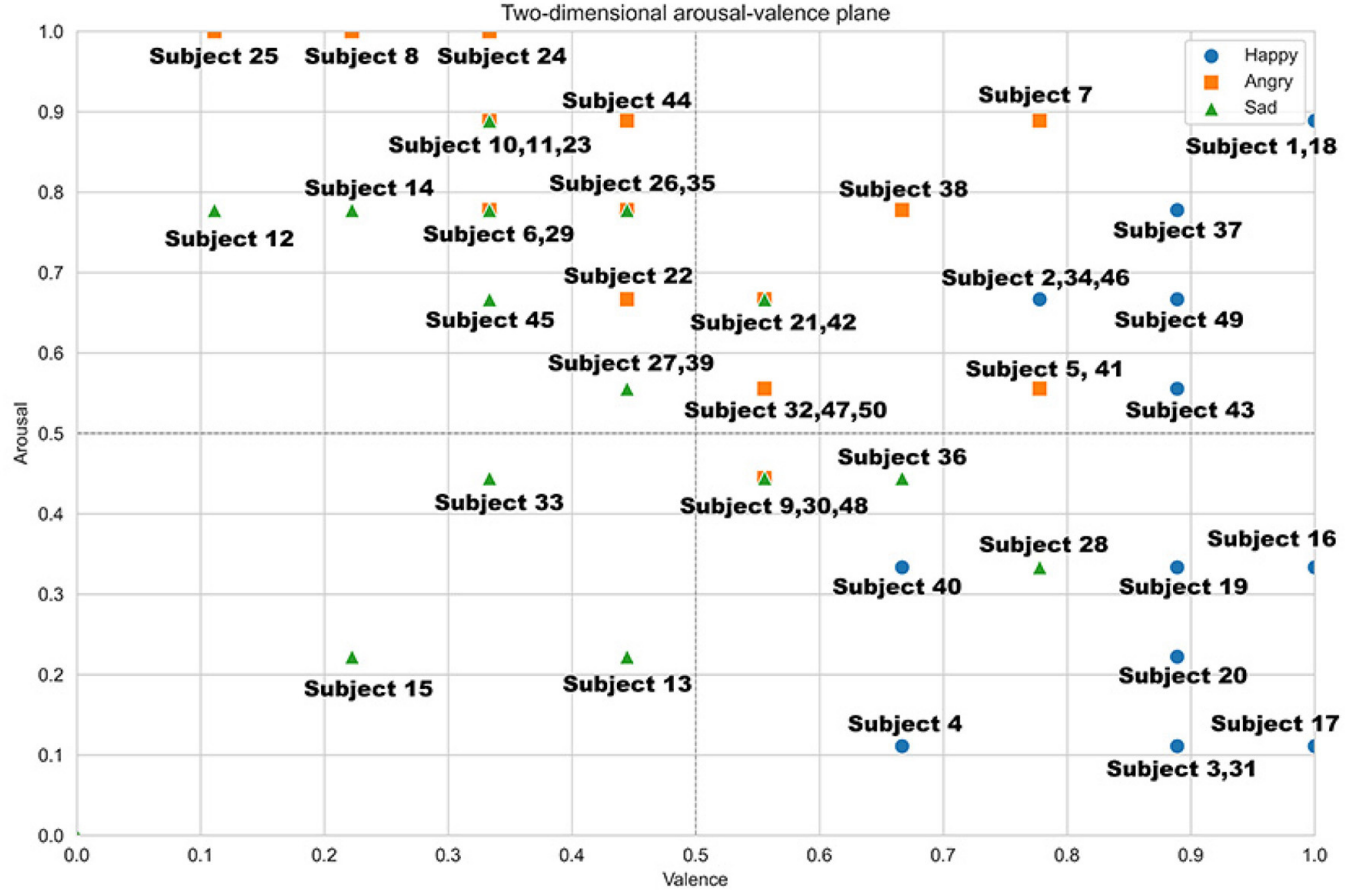
Emotional characterization of the 50 participants of the continuous model in the two-dimensional excitation-valence plane.

Figure 6 shows the distribution of the 50 participants after normalization of their SAM test results. Since the emotional states are diverse and each corresponds to a specific region in the two-dimensional plane (based on arousal and valence), this research categorized the regions where the three target emotions are found: happiness, anger, and sadness. If a participant's induced emotion, based on their arousal-valence values, fell within the expected region for the emotion, their data were considered valid.

Based on the results obtained, among the 50 participants subjected to emotion induction tests in a simulated driving environment, only 42% of the induced emotions matched the actual emotions experienced. This equated to 21 participants in total, with 9 indicating happiness, 9 indicating anger, and 3 indicating sadness. The remaining 58% of participants did not match the induced emotion with the actual one. The dataset contains information from 21 participants, including motor activity data (throttle pedal movement, brake pedal movement, and steering wheel angle) totaling 302,626 records (Neutral = 67,462, Happy = 99,148, Angry = 70,608, Sad = 65,408).

Additionally, visual data (images) collected from the participants' facial geometry totaled 3,361 (Neutral = 1,464, Happy = 1,037, Angry = 366, and Sad = 494), based on the discrete emotional model proposed by Paul Ekman. This research combined discrete and continuous emotional models to assess emotions, based on the premise that facial expressions do not always fully reflect the participant's emotional state, as suggested by Ekman. Therefore, the arousal-valence model proposed by James Russell was also used. The emotion prediction process characterized dimensional emotion labels using both continuous and discrete representations. Recent studies, such as Mihalache and Burileanu (2021), have shown performance gains when converting continuous labels into a discrete set, despite some label quantization error. AlBadawy and Kim (2018) demonstrated the effectiveness of using joint representations of discrete and continuous emotions in describing dynamically changing affective behavior.

Given this, the present study induced emotional states, characterized them using the continuous model, and verified that the induced emotions matched the actual ones via the SAM tool. Next, the participant's visual data were analyzed to ensure that their facial expressions aligned with the expected outcomes in the discrete model. Although the two models differ, the data collection procedure for each emotional state was as follows: if a specific emotion, such as happiness, was induced and matched the real emotion, and if the participant's arousal-valence values fell within the corresponding range (e.g., 6–9 for both valence and arousal), the motor data and corresponding facial geometry data were considered valid for that emotion.

**Figure 7:**
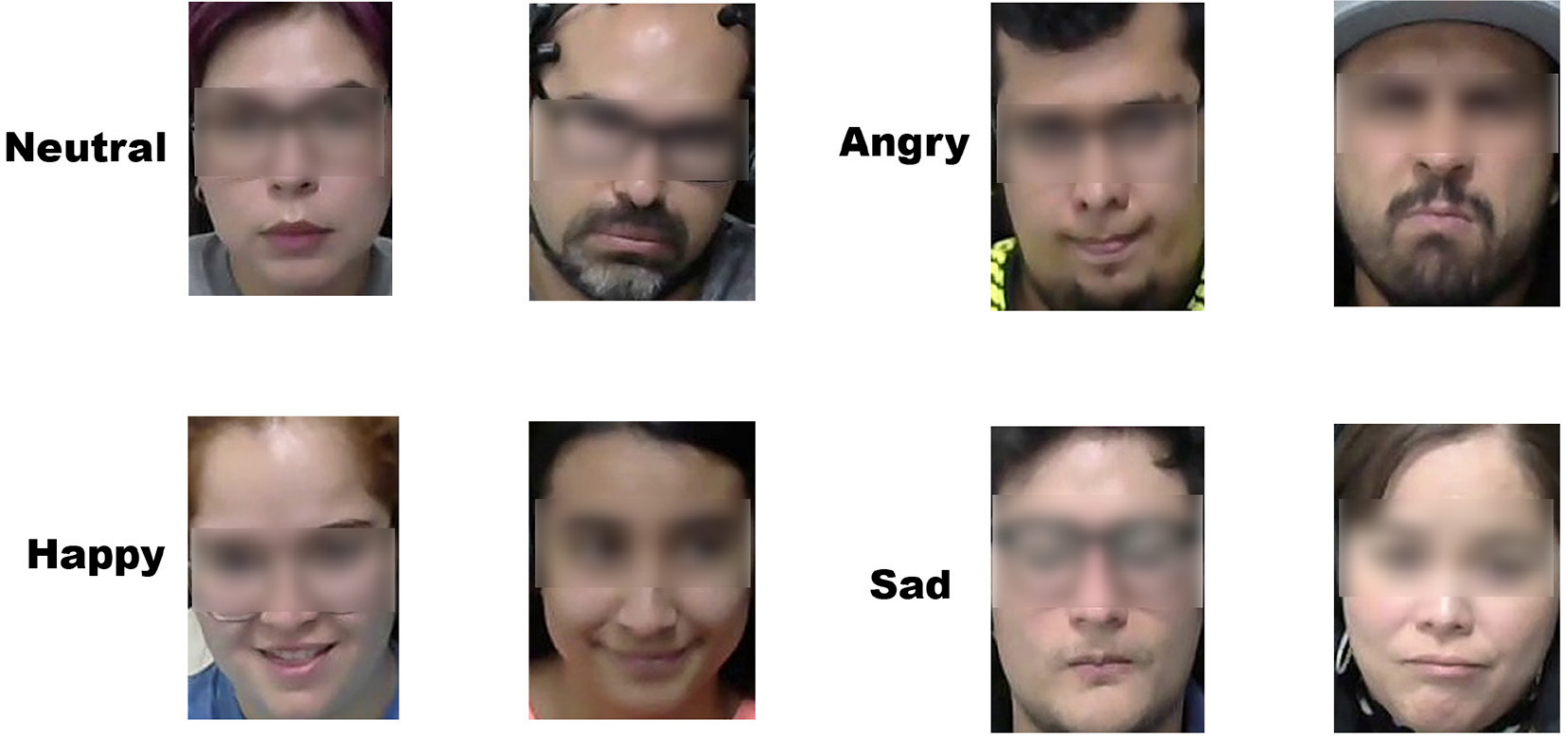
Sample images of the four emotion categories of the generated dataset.

Paul Ekman states that there are six basic emotions universally expressed by humans in response to psychological triggers. For the neutral emotion, images that did not fall into any of the six basic emotions were collected. Figure 7 presents images showing the discrete emotional characterization of the neutral, happy, angry, and sad states during the driving tests.

**Figure 8:**
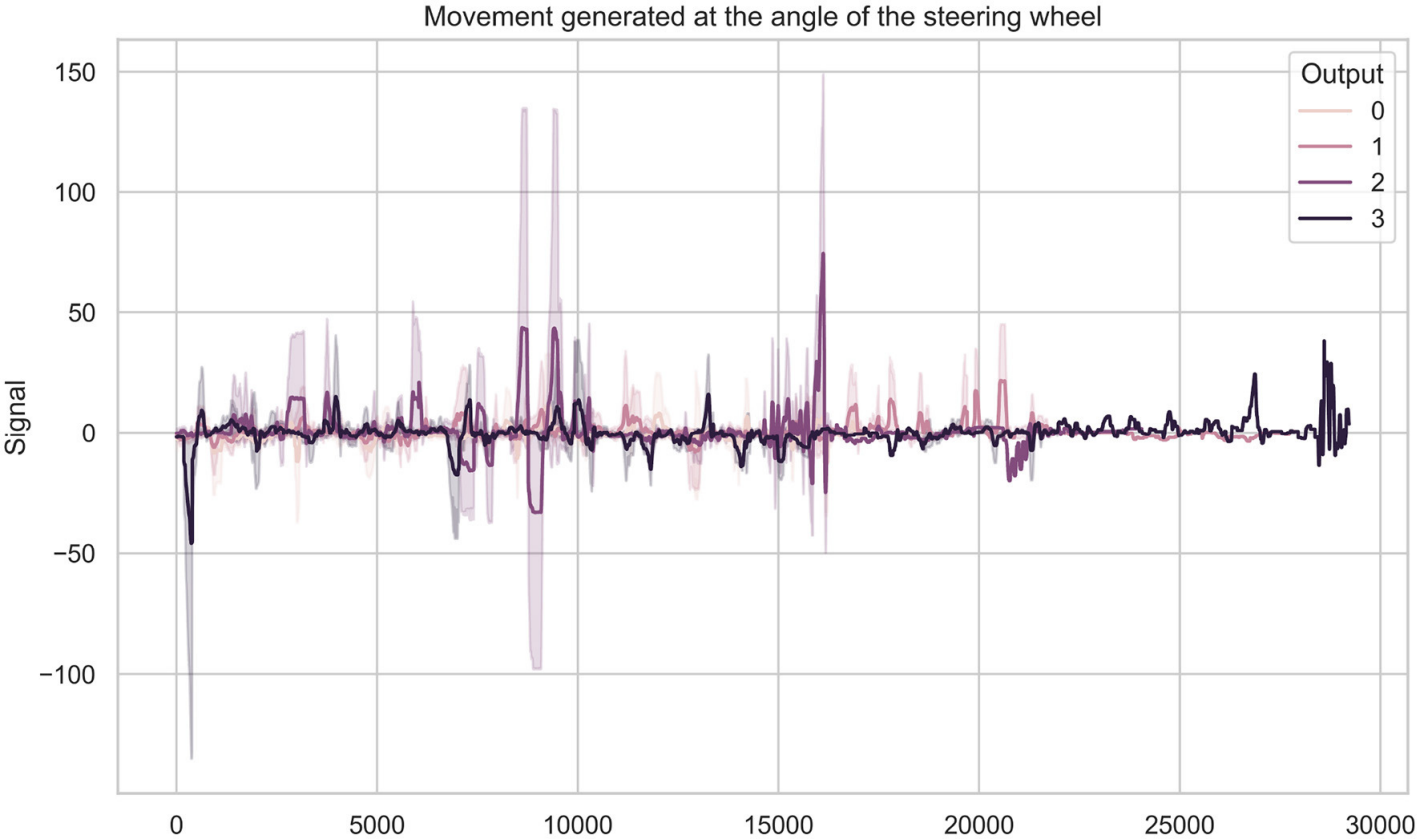
Steering wheel angle signal in different emotional states. Where each numerical value represents an emotion 0 for neutral, 1 for happy, 2 for angry, and 3 for sad.

After collecting and constructing the dataset, an in-depth analysis of motor activity was performed. Human behavior is complex and constantly changing, so it was essential to study drivers' behaviors across different emotional states while interacting with basic driving elements. Figure 8 illustrates the signals generated from steering wheel angle measurements in different emotional states, visually demonstrating variations in motor activity.

Windows of 50 records were analyzed and processed in steps of 5 from motor activity data to identify segments that provide relevant information for recognizing motor activity associated with the target emotional states. While various methods, techniques, algorithms, and transforms can perform these complex tasks, implementing feature selection through machine learning algorithms proved to be an effective tool for identifying key data related to certain emotions. Feature selection is essential for the successful application of machine learning and data mining algorithms in real-world scenarios. Numerous methods for relevant feature selection have been proposed in the literature, including RFE, which reduces irrelevant, redundant features, noisy data, and high dimensionality (Jeon and Oh, 2020).

The process involved applying the RFE technique with different machine learning algorithms using the motor activity dataset. Eighty percent of the data were used for training, and the remaining 20% for blind testing. Table 2 shows the performance of the models in terms of accuracy, which evaluates how often the model correctly predicts outcomes (Pacurari et al., 2023). Precision was also used, measuring how often the model correctly predicts true positives relative to the total number of positive predictions made (Imani and Arabnia, 2023). Additionally, recall (or sensitivity) was employed to measure how often the model correctly identifies true positives from all positive samples in the dataset. Lastly, the F1-Score, a metric derived from precision and recall, was used to assess the models' performance. F1-scores range from 0 to 1, with higher values indicating better model performance (Imani and Arabnia, 2023).

**Table 2:** Results obtained from emotion classification models using different learning algorithms and motor activity as data source.

**Algorithm**	**Emotion**	**Precision**	**Recall**	**F1-Score**
RFC	Neutral	0.91	0.90	0.90
	Happy	0.88	0.90	0.89
	Angry	0.89	0.88	0.88
	Sad	0.89	0.88	0.88
**Accuracy**				**0.89**
DT	Neutral	0.80	0.82	0.81
	Happy	0.80	0.79	0.80
	Angry	0.79	0.79	0.79
	Sad	0.77	0.76	0.76
**Accuracy**				**0.79**
ABC	Neutral	0.42	0.37	0.40
	Happy	0.42	0.50	0.46
	Angry	0.57	0.52	0.54
	Sad	0.44	0.40	0.42
**Accuracy**				**0.46**
LDA	Neutral	0.20	0.08	0.11
	Happy	0.33	0.83	0.47
	Angry	0.44	0.14	0.21
	Sad	0.26	0.02	0.03
**Accuracy**				**0.32**

The bold values represents the overall accuracy of each of the implemented machine learning algorithms.

Based on the results, the model generated by the Random Forest Classifier (RFC) algorithm achieved the best performance in identifying motor activity segments that best characterize target emotional states, achieving 89% accuracy.

As a result of this procedure, 9 significant motor activity features were identified: 5 related to steering wheel angle, 0 related to brake movement, and 4 related to throttle movement. By identifying key signal segments that improve the final emotion recognition model, driving behavior could be distinguished across different emotional states. For example, the behavior related to steering wheel movement was significantly more intense during anger than for other emotions. Similarly, the neutral emotion also showed greater steering wheel movement, though not as intense as the anger emotion.

Although accelerator pedal movement signals seemed to fluctuate within similar ranges, there were notable differences between emotions. In particular, anger showed the highest levels of accelerator pedal engagement, indicating that participants pressed harder on the pedal when feeling angry.

The CNN architectures, VGG16 and VGG19, demonstrated the highest potential in recognizing emotions from the 3,361 images of drivers in various emotional states, achieving accuracy rates of 98% and 99%, respectively. These models generated probability vectors between 0 and 1 for each image, which were subsequently integrated with the engine dataset that had been correctly classified using the RFE algorithm. In the case of the Inception V3 network, promising results were also obtained, with an accuracy of 97.2%. However, the accuracy and loss plots for Inception V3 did not display as stable behavior as those for the VGG networks. According to Pathar et al. (2019), for a model to be validated satisfactorily–in this case, emotion recognition–the validation loss should be similar to or greater than the training loss. If the validation loss is lower than the training loss, the model may be underfitted and should be trained for more epochs. In the models generated using VGG16 and VGG19, this condition was satisfied, as the validation loss followed a similar pattern to the training loss. Finally, EfficientNet achieved an accuracy of only 43.5%, leading to its early dismissal from further consideration.

After identifying the most relevant motor activity features and reducing redundancy and dimensionality through feature selection, as well as extracting probability vectors for facial expressions using deep learning, a mid-fusion technique was implemented. This approach was necessary due to the distinct nature of the data: motor signals (such as steering wheel angle, pedal movement, and braking data) are inherently time series and require specialized feature extraction or vectorization methods, whereas facial data, typically extracted from images or video frames, require CNNs to identify relevant geometric patterns associated with emotions. These two data types differ significantly in their structures and processing requirements. Mid-fusion enables each modality to be processed independently, using feature extraction techniques specifically tailored to the characteristics of each data type, preserving the unique features of each modality prior to fusion (Hassani et al., 2024).

In contrast, early fusion involves combining raw data from different modalities at the initial stage. While this approach can integrate data quickly, it may result in information overload and make it difficult for the model to extract meaningful features from each modality. This is especially true when raw data from different formats (e.g., image data vs. time-series data) are combined. Early fusion often requires significant preprocessing to handle discrepancies between modalities, potentially diminishing the richness of features that can be learned (Gadzicki et al., 2020).

On the other hand, mid-fusion allows for independent feature extraction from each modality (e.g., probability vectors for facial emotions and key motor activity features). This preserves the unique characteristics of each modality and facilitates a more meaningful integration of information at a higher level of abstraction, where patterns between the modalities can be more effectively recognized.

Late fusion, in which decisions are made independently for each modality and then combined, may fail to capture cross-modal interactions, as each modality is treated in isolation before fusion (Boulahia et al., 2021). By fusing at an intermediate stage (mid-fusion), the model can capture relationships between motor activity and facial expressions, leading to a more nuanced and effective emotion recognition system.

In this approach, facial data were processed using a CNN to extract geometric facial features, while motor activity data were analyzed using feature selection algorithms (such as random forests, decision trees, etc.). This fusion methodology ensures that the most relevant features from each domain are incorporated into the final model for multimodal emotion recognition, which was built using a one-dimensional convolutional neural network (1D-CNN). The hyperparameters for this model are presented in Table 3.

**Table 3:** 1D-CNN architecture hyperparameters for training.

**Hyperparameter**	**1D-CNN**
Input	1 X 13
Activation functions	softplus
Epochs	500
Optimizer	Adam
Convolutional layers	5
Kernels	64,128,256,512,1024
Loss	sparse categorical crossentropy
Output function	softmax
Number of classes	4
Dense layers	4 of 512,256,128,64 neurons
Batch size	32

With the architecture and hyperparameters of the one-dimensional convolutional neural network (1D-CNN) defined, we conducted a k-fold cross-validation process. This method involves dividing the dataset into k subsets, as described by Wong and Yeh (2020). Each subset takes a turn serving as test data, while the remaining subsets are used for training. The validation process runs for k iterations, corresponding to the number of folds, so that each fold is used as test data exactly once. To determine the model's overall performance, we calculate the arithmetic mean of the results from all iterations. In this study, we selected k = 5, which is a common choice for k-fold validation as it balances computational efficiency and robust model evaluation. The k-fold validation method is widely regarded as a robust way to assess the effectiveness of classification models, as it partitions the dataset and treats each group as an independent validation set (Wong and Yeh, 2020). The results of the k-fold cross-validation are presented in Table 4.

**Table 4:** Classification results per k-fold for multimodal emotion recognition.

**K**	**Emotion**	**Precision**	**Recall**	**F1-score**
1	Neutral	1.00	1.00	1.00
	Happy	1.00	1.00	1.00
	Angry	0.98	0.99	0.98
	Sad	0.96	0.95	0.95
**Accuracy**				**0.99**
2	Neutral	1.00	1.00	1.00
	Happy	1.00	1.00	1.00
	Angry	1.00	0.99	0.99
	Sad	0.97	1.00	0.98
**Accuracy**				**1.00**
3	Neutral	1.00	1.00	1.00
	Happy	1.00	1.00	1.00
	Angry	0.99	0.98	0.98
	Sad	0.94	0.96	0.95
**Accuracy**				**0.99**
4	Neutral	1.00	1.00	1.00
	Happy	1.00	1.00	1.00
	Angry	0.75	1.00	0.85
	Sad	0.00	0.00	0.00
**Accuracy**				**0.85**
5	Neutral	1.00	0.96	0.98
	Happy	1.00	1.00	1.00
	Angry	1.00	0.98	0.99
	Sad	0.92	0.99	0.95
**Accuracy**				**0.99**
Overall classification report	Neutral	1.00	0.99	1.00
	Happy	1.00	1.00	1.00
	Angry	0.93	0.99	0.96
	Sad	0.95	0.78	0.85
**Overall accuracy**				**0.96**

The bold values represents the accuracy obtained in each K-fold.

**Figure 9:**
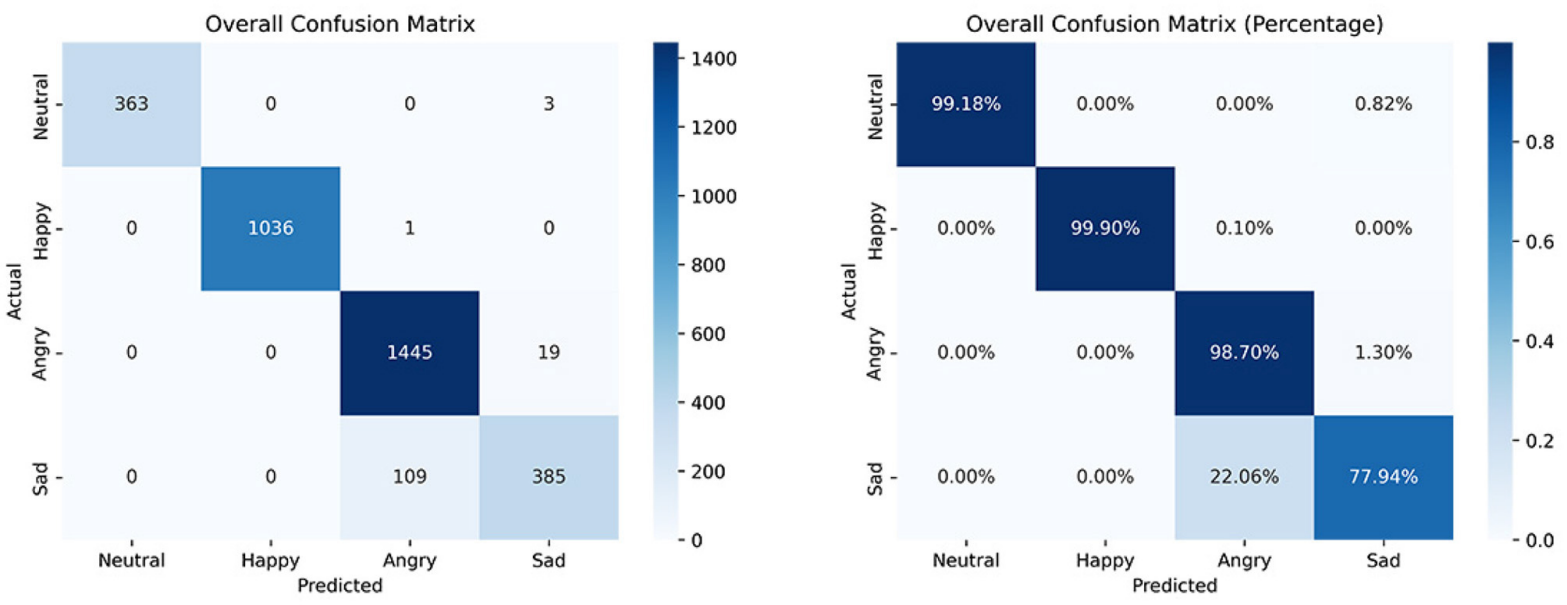
Confusion matrices resulting from the multimodal emotion recognition model. On the left side is shown the matrix with the number of correctly classified observations and on the right side the percentage of correctly classified observations.

A confusion matrix is a tabular representation of the actual labels versus the model's predictions, providing insight into the performance of the model. Each row of the confusion matrix represents the instances predicted to belong to a specific class, while each column represents the actual instances from the dataset. This matrix serves as the foundation for calculating other key performance metrics (Heydarian et al., 2022). Figure 9 shows the confusion matrices generated for classifying the target emotions identified in this research. Of the 361 integrated motor activity datasets combined with probability vectors from facial expression recognition, the 1D-CNN model correctly identified 363 instances of neutral emotion, 1,036 of happiness, 1,445 of anger, and 385 of sadness.

**Figure 10:**
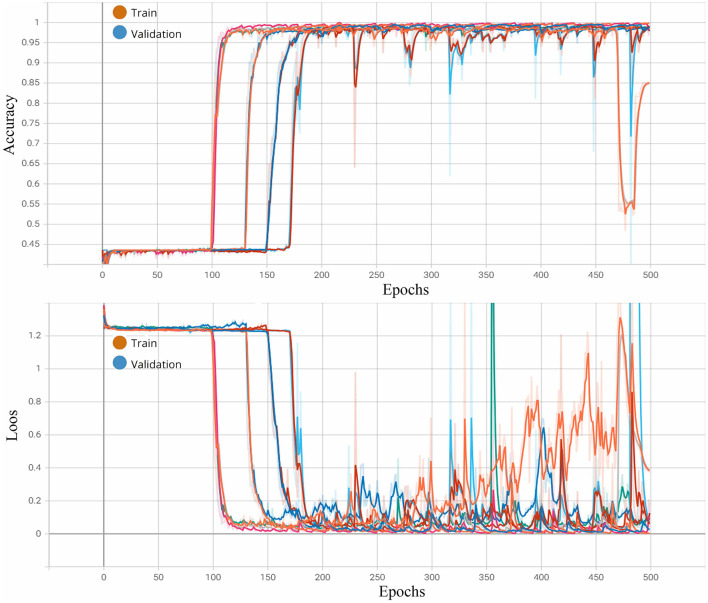
Loss and accuracy curves over 500 epochs during the training and validation of the multimodal emotion recognition model.

Figure 10 illustrates the network's performance in processing multimodal data over 500 epochs for each fold using the 1D-CNN. As seen in the training process, the accuracy and loss curves were consistently aligned, indicating stable model performance across epochs. This confirms that overfitting was not an issue, affirming the network's ability to generalize in recognizing emotions across the dataset.

Finally, an analysis of the computational complexity of the model was carried out. Since the model is intended for real-time inference in automotive systems, it is crucial to address memory usage, processing time and scalability. As a result, the following results were obtained 383.87 MB of memory usage, with an average processing time of 0.50 s. For real-time models, it is important to see how the model responds to multiple simultaneous requests. Simulations of 100 concurrent inference requests were performed to measure how the response time changes, with an average of 32.37 s.

The results obtained in this section are highly significant, demonstrating the model's ability to accurately identify four universal emotions (neutral, happiness, anger and sadness) in the discrete model, as well as three emotions in the continuous model, reaching a statistically significant accuracy of over 90%. This innovative methodology represents a valuable advance in the field of affective computing in driving environments.

However, it is important to note that the inference time of the model needs improvement. Accurately recognizing emotions in real time is crucial because emotions can be brief and subject to rapid change. Optimizing the inference time would improve the system's ability to respond effectively to these momentary emotional changes.

## 5 Discussion

There are several studies with promising approaches to emotion recognition in drivers that have achieved statistically significant results in terms of accuracy. However, studies focusing on multimodal artificial intelligence, which essentially involves processing and understanding information from different sources, have been less explored in driving environments. Table 5 presents a comparison between state-of-the-art methods and the proposed methodology, examining the data sources, algorithms used, and the performance achieved.

**Table 5:** State-of-the-art, data source, algorithms and accuracy for emotion recognition.

**Author**	**Data source**	**Algorithm**	**Accuracy**
Lee et al. (2018)	Facial Geometry	CNN	99.95%
Verma and Choudhary (2018a)	Facial Geometry	CNN	98.80%
Patil and Veni (2019)	Facial geometry	SVM	86.70%
Shafaei et al. (2019)	Multimodal	SVM	94.00%
Wang et al. (2020)	ECG	Artificial neural network	91.11%
Cui et al. (2020)	Facial geometry	CNN	83.10%
Naqvi et al. (2020)	Facial geometry	CNN	98.93%
Du et al. (2021)	Multimodal	CNN	84.32%
Oh et al. (2021)	Multimodal	CNN	86.80%
Xiao et al. (2022)	Facial geometry	CNN	97.20%
Zaman et al. (2022)	Facial geometry	CNN	97.91%
Sukhavasi et al. (2022)	Facial geometry	CNN and SVM	94.09%
Mou et al. (2023)	Mutimodal	CNN	85.34%
Hieida et al. (2023)	Mutimodal	Sparse logistic regression	67.00%
Hu et al. (2024)	Mutimodal	Multitask learning network	67.92%
**Our method (2023)**	**Mutimodal**	**CNN**	**96.00%**

The bold values represents the overall accuracy obtained from the multimodal emotion recognition model in drivers proposed in this research.

Training CNNs with images has yielded significant results for driver emotion recognition, as shown in the table. However, as mentioned in the problem statement, accurately identifying emotions through constant monitoring of drivers presents limitations, primarily due to occlusion caused by various factors. Current research, such as that by Mou et al. (2023), combines large datasets related to both the driver and the surrounding environment. This research demonstrated that using data from the driver's eyes, vehicle data, and the environment across different scenarios to generate recognized emotions can be processed through a ConvLSTM network with an accuracy exceeding 80%. However, the model still relies heavily on accurate visual data from drivers in real time to achieve these levels of accuracy, which brings back the same issues found in methodologies based on video capture devices.

This limitation highlights the importance of multimodal modeling approaches, which address such problems by incorporating alternative data sources that either relieve or complement image analysis systems. These models have also proven to be highly relevant in the field of driver emotion recognition, demonstrating efficient performance.

The work presented by Shafaei et al. (2019) demonstrates the feasibility of classifying emotions using vehicle parameters generated by 16 participants, along with facial expressions from datasets such as CK+ and JAFFE, using SVM as a classifier. However, the limited potential of these traditional machine learning techniques may impede their real-world application. Despite this, their findings align with this study in a critical way: drivers tend to exhibit more active and abrupt behaviors when they are angry, happy, or excited. Conversely, their driving becomes more passive with fewer eye or body movements when they are tired or sad. These behavioral patterns correlate with emotional categories in the continuous model. Although the study is relevant for emotion identification, it becomes somewhat outdated due to the lack of more sophisticated AI algorithms like CNNs, which are superior tools for creating models that can be implemented in real-world settings.

Similarly, Du et al. (2021) proposes identifying emotions through heart rate and facial features from 16 volunteers in a simulated environment, yielding promising results. However, current devices for acquiring cardiac data can be invasive for drivers, leading to poor ergonomics and frequent emotional disturbances. Despite these limitations, their results demonstrate the ability of their methodology to recognize the emotions studied using convolutional neural networks (CNNs). Although the study lacks concrete details on the emotion induction process, it is inferred that participants assumed various emotional roles and behaviors. This suggests a subjective emotion labeling process, which limits the methodology's applicability to other scenarios. Moreover, the study did not base its work on an emotion theory, making its recognition approach less objective and precise than this research.

In contrast, Oh et al. (2021) developed a multimodal model combining facial images and dermal activity data from 13 volunteers (six men and seven women). This less invasive method could potentially be integrated into vehicle manufacturing. However, its performance is suboptimal compared to the results of this study. One key difference is the emotion induction techniques used. The authors applied methods such as movie watching and passage writing, which are effective but limited to generating emotions during the activity itself. In contrast, autobiographical memory techniques–used in this study–work well because participants recall emotional moments from their lives while driving. Additionally, the study's sampling frequency (10 Hz) was higher than the 5 Hz used in this research. The lower frequency, combined with AI-based selection of samples from the continuous motor activity signal, reduced noise and significantly improved recognition model performance.

Among the studies closely related to this research that include data beyond facial geometric changes, Hieida et al. (2023) fused several physiological signals from drivers using Sparse Logistic Regression (SLR) on multimodal data to recognize negative emotions. They achieved a 74.0% area under the curve (AUC) for successful emotion classification, integrating the results into an ADAS system for greater driver comfort. However, their proposal's performance is still much lower compared to other studies, including this one. The use of SLR, while simpler and faster with lower computational requirements, is less effective for this type of data compared to more complex algorithms like CNNs. CNNs generally provide higher performance due to their ability to handle multimodal signals and automatically extract relevant features. Additionally, the ability of motor activity alone to recognize different emotional patterns has been demonstrated, further validating this study's findings.

The current proposal by Hu et al. (2024) demonstrated the potential of facial videos and driver behavior (brake pedal force, Y-axis position, and vehicle Z-axis position) as inputs in a multitask training approach. However, not considering other elements of this second data source significantly affects model performance, an important aspect that the present study does consider to improve accuracy in emotion identification. Also, the process that is carried out is more complex compared to the one presented, which could result in a longer inference time in real time.

Despite the various approaches discussed, most of the efforts have focused on image-based models that utilize deep learning algorithms like CNNs for emotion recognition. However, Vision Transformers (ViTs) are gaining recognition as an effective alternative to CNNs for various vision tasks, as they have been shown to be more robust against image distortions. ViTs take a different approach by exploring topological relationships between image patches, allowing them to capture more global and far-reaching connections, although they require more data-intensive training (Dai et al., 2021). ViT performance also relies heavily on factors like optimizer selection, dataset-specific hyperparameters, and network depth, more so than CNNs. Preprocessing with overlapping convolutional filters of smaller size (stride < size) has been shown to contribute to performance and stability (Xiao et al., 2021).

CNNs, in contrast, deliver outstanding results even with relatively smaller datasets, compared to the larger datasets required by ViTs. This performance difference is largely attributed to the different inductive biases inherent to each architecture. CNNs' filter-based structure allows for quick identification of specific image features, but this same architecture limits their ability to capture more complex global relationships (Raghu et al., 2021).

This study leverages all the intrinsic properties of CNNs to address the challenges identified in the literature. By focusing on data related to drivers' interactions with vehicle elements, primarily the steering wheel, accelerator, and brake, this study demonstrates superior performance compared to state-of-the-art multimodal models that rely on motor activity data. The methodology presented here achieves higher performance due to the factors discussed throughout this section.

## 6 Conclusions

In conclusion, this study aimed to contribute with a new and innovative model for emotion recognition in drivers through motor activity data and facial expressions, implementing deep learning techniques such as CNNs, where it was possible to obtain an accuracy of 98.0% by giving equal weight to motor information and facial geometry data using feature selection algorithms to avoid outliers, redundancy and performance loss. These results demonstrate a high viability of the model for implementation in real environments, in addition to filling some gaps found in current studies based on cameras and driver behavior, where the performance of the presented proposal competes with any model developed to date.

A significant contribution was also made as a first approximation to the technique of augmented autobiographical memory as a method of inducing emotions in drivers, offering precise figures of success of the process in a specific experimental environment, guiding researchers to explore other options for inducing emotions in simulated driving environments.

It is essential to mention that this type of proposals could potentially help reduce the number of accidents related to negative emotions, since these emotions are among the main psychological factors that can influence driving behavior (Maldonado et al., 2020; Šeibokait et al., 2017). This study also concluded that different emotional states effectively cause a change in driving behavior and style. This allows us to identify emotions through the interaction we have with the vehicle. In addition, this type of proposals could improve the user experience, provided that vehicle systems know the emotions of drivers in real time, giving rise to more appropriate and personalized systems for each individual.

On the other hand, the study of motor activity is not limited to the automotive industry, but could also be extended to everyday life, where various emotions could be identified from data generated by mobile devices such as smartphones, smart watches and other smart devices for daily use. Although there are still different sources of information and emotions related to them, this study represents an important contribution as a first approximation to the recognition of emotions in drivers, with the aim of improving the quality and safety of transport systems.

### 6.1 Future work

As future work, it is proposed to deepen in different methodologies to neutralize and induce emotions in drivers, since one of the major limitations of the present study is the low level of participants who were successfully induced an emotion through the SAM tests, carried out in experimental simulated driving environments. Nevertheless, these results offer guidance, cautions and limitations of the technique in case we wish to implement it in future research. In addition, it is suggested to expand the group of participants to ensure a broader spectrum of emotions and related motor activity and facial geometry data. It is also proposed to explore other scenarios such as drowsiness, drunkenness and distraction, where this model could potentially be applied, in order to mitigate traffic accidents. Even the validation process could be extended, particularly with testing under different conditions. This extension will improve the robustness and applicability of the emotion recognition model. Nevertheless, the present research establishes a first approach for real-time emotion recognition.

## Data Availability

The raw data supporting the conclusions of this article will be made available by the authors, without undue reservation.
